# The *FUT2* Variant c.461G>A (p.Trp154*) Is Associated With Differentially Expressed Genes and Nasopharyngeal Microbiota Shifts in Patients With Otitis Media

**DOI:** 10.3389/fcimb.2021.798246

**Published:** 2022-01-14

**Authors:** Christina L. Elling, Melissa A. Scholes, Sven-Olrik Streubel, Eric D. Larson, Todd M. Wine, Tori C. Bootpetch, Patricia J. Yoon, Jennifer M. Kofonow, Samuel P. Gubbels, Stephen P. Cass, Charles E. Robertson, Herman A. Jenkins, Jeremy D. Prager, Daniel N. Frank, Kenny H. Chan, Norman R. Friedman, Allen F. Ryan, Regie Lyn P. Santos-Cortez

**Affiliations:** ^1^ Department of Otolaryngology-Head and Neck Surgery, School of Medicine, University of Colorado Anschutz Medical Campus, Aurora, CO, United States; ^2^ Human Medical Genetics and Genomics Program, University of Colorado Anschutz Medical Campus, Aurora, CO, United States; ^3^ Department of Pediatric Otolaryngology, Children’s Hospital Colorado, Aurora, CO, United States; ^4^ Division of Infectious Diseases, Department of Medicine, School of Medicine, University of Colorado Anschutz Medical Campus, Aurora, CO, United States; ^5^ Division of Otolaryngology, Department of Surgery, San Diego School of Medicine and Veterans Affairs Medical Center, University of California, La Jolla, CA, United States; ^6^ Center for Children’s Surgery, Children’s Hospital Colorado, Aurora, CO, United States

**Keywords:** *FUT2*, microbiota, otitis media, p.Trp154*, RNA-sequencing, rs601338

## Abstract

Otitis media (OM) is a leading cause of childhood hearing loss. Variants in *FUT2*, which encodes alpha-(1,2)-fucosyltransferase, were identified to increase susceptibility to OM, potentially through shifts in the middle ear (ME) or nasopharyngeal (NP) microbiotas as mediated by transcriptional changes. Greater knowledge of differences in relative abundance of otopathogens in carriers of pathogenic variants can help determine risk for OM in patients. In order to determine the downstream effects of *FUT2* variation, we examined gene expression in relation to carriage of a common pathogenic *FUT2* c.461G>A (p.Trp154*) variant using RNA-sequence data from saliva samples from 28 patients with OM. Differential gene expression was also examined in bulk mRNA and single-cell RNA-sequence data from wildtype mouse ME mucosa after inoculation with non-typeable *Haemophilus influenzae* (NTHi). In addition, microbiotas were profiled from ME and NP samples of 65 OM patients using 16S rRNA gene sequencing. In human carriers of the *FUT2* variant, *FN1, KMT2D, MUC16* and *NBPF20* were downregulated while *MTAP* was upregulated. Post-infectious expression in the mouse ME recapitulated these transcriptional differences, with the exception of *Fn1* upregulation after NTHi-inoculation. In the NP, Candidate Division TM7 was associated with wildtype genotype (FDR-adj-*p*=0.009). Overall, the *FUT2* c.461G>A variant was associated with transcriptional changes in processes related to response to infection and with increased load of potential otopathogens in the ME and decreased commensals in the NP. These findings provide increased understanding of how *FUT2* variants influence gene transcription and the mucosal microbiota, and thus contribute to the pathology of OM.

## Introduction

Infection and inflammation of the middle ear (ME), known as otitis media (OM), is the most frequently diagnosed disease in infants and young children in the United States and is globally a leading cause of hearing loss (Monasta et al., 2012; GBD, 2021). In children, an estimated 60% of hearing loss is due to preventable causes, and infections and chronic OM account for around 31% of pediatric hearing loss (Schilder et al., 2016; GBD, 2021). In the United States, treatment of OM costs over $5 billion annually and typically includes antibiotics and surgery such as tympanostomy tube insertion (Schilder et al., 2016; Suaya et al., 2018). OM risk and pathology are influenced by many factors including environmental factors such as age, sex, daycare attendance and breastfeeding as well as genetic factors (Zhang et al., 2014; Brennan-Jones et al., 2015). Heritability of OM is estimated to be as high as 74%; furthermore, genes related to OM predisposition are known to function in pathways that include innate immune response, cell-mediated immune dysfunction and pathogen-host-environment interactions (Casselbrant et al., 1999; Mittal et al., 2014).

OM is often bacterial or viral in origin, wherein pathogens in the nasopharynx (NP) migrate *via* the Eustachian tube to the ME. This creates an inflammatory cycle in the ME with an accumulation of mucus and fluid which can lead to permanent damage and hearing loss (Rosenfeld et al., 2013). It is important to note that prior to infection, the ME is essentially sterile as it is generally separated from the external environment by the tympanic membrane, whereas the NP has an established microbiota that can vary based on microbial exposure and host genetics, but these microbes in the NP do not become resident in the ME if the Eustachian tube is functioning well (Jervis-Bardy et al., 2019). Some NP commensals are potential opportunistic otopathogens of the ME (Yatsyshina et al., 2016). It is well-known that increased abundance of potential otopathogens in the NP is associated with higher risk for OM (Jervis-Bardy et al., 2017; Browne et al., 2021; Xu et al., 2021).

*FUT2* (MIM 182100) encodes alpha-(1,2)-fucosyltransferase which is responsible for secretion and expression of ABO(H) antigens on mucosal epithelia (Kelly et al., 1995). Secretory status directly influences pathogen binding in mucosal epithelia in multiple organ systems. The *FUT2* stop variant c.461G>A (p.Trp154*; rs601338) has been associated with multiple mucosal phenotypes and is in strong linkage disequilibrium (LD) with a synonymous *FUT2* variant rs681343 that was previously associated with childhood ear infections in genome-wide association studies (GWAS) (Pickrell et al., 2016; Tian et al., 2017). This variant has also been confirmed to confer familial OM risk in multiple cohorts (Santos-Cortez et al., 2018). Additionally, *Fut2* expression transiently increased in the mouse ME after infection with non-typeable *Haemophilus influenzae* (NTHi), which is a common otopathogen in humans (Santos-Cortez et al., 2018).

Non-secretors, i.e., homozygous for *FUT2* c.461G>A, show higher rates of bacterial infections [e.g. with *Streptococcus pneumoniae*, NTHi in different organ systems], but decreased susceptibility to viral infection (i.e. viral diarrhea or HIV-1), possibly due to the effects of the glycan on the mucus barrier (Magalhaes et al., 2016; Azad et al., 2018; Santos-Cortez et al., 2018). Though *FUT2* is well-studied, to our knowledge there are no previous studies of transcriptome-wide differences in host gene expression based on carriage of the *FUT2* c.461G>A variant in humans. Furthermore, to date only seven studies investigated changes in the host microbiota that were associated with carriage of this variant (Rausch et al., 2011; Wacklin et al., 2011; Wacklin et al., 2014; Kumar et al., 2015; Davenport et al., 2016; Kumbhare et al., 2017; Turpin et al., 2018; Chen et al., 2021). These studies were limited to assessment of the gut microbiota according to variant carriage and identified associations seemed to be environment- or disease- specific. While some studies observed no associations between gut microbiome and *FUT2* c.461G>A genotype, others noted that *Bifidobacterium* levels, among other taxa, were significantly different between variant carriers and wildtype (Wacklin et al., 2011; Wacklin et al., 2014; Davenport et al., 2016; Turpin et al., 2018). Furthermore, in Crohn’s Disease and throughout pregnancy, the *FUT2* c.461G>A variant was associated with differences in the gut microbiota diversity and abundance of individual taxa (Rausch et al., 2011; Kumar et al., 2015).

In order to further elucidate the role of *FUT2* in OM pathogenesis, the goal of this study was to investigate the potential downstream effects of the *FUT2* c.461G>A (p.Trp154*) variant on gene expression and site-specific colonization by commensals and known otopathogens. Characterization of this common variant and its role in the interplay between host genetics, host immune response, and mucosal microbiotas not only expands our general understanding of these complex relationships but also, within the context of OM, provides clinically relevant insight that can be used to better determine individual risk and inform treatment. In this study, we performed differential expression (DE) analysis on RNA-sequence data from saliva of OM-affected individuals and identified multiple differentially expressed genes based on carriage of the *FUT2* c.461G>A variant. These DE genes were replicated using genome-wide expression data from infected mouse ME. We also performed microbiota analysis using 16S rRNA sequence data from ME and NP samples of OM-affected individuals and identified bacterial taxa that were different in relative abundance according to genotype.

## Materials and Methods

### Ethics Approval

Ethical approval was obtained from the COMIRB prior to the start of the study. Informed consent was obtained from study participants, including parents of children enrolled in the study. The IACUC of the Veterans Affairs Medical Center, San Diego, California granted approval for mouse studies.

### Subject Ascertainment and Sample Collection

**Figure 1:**
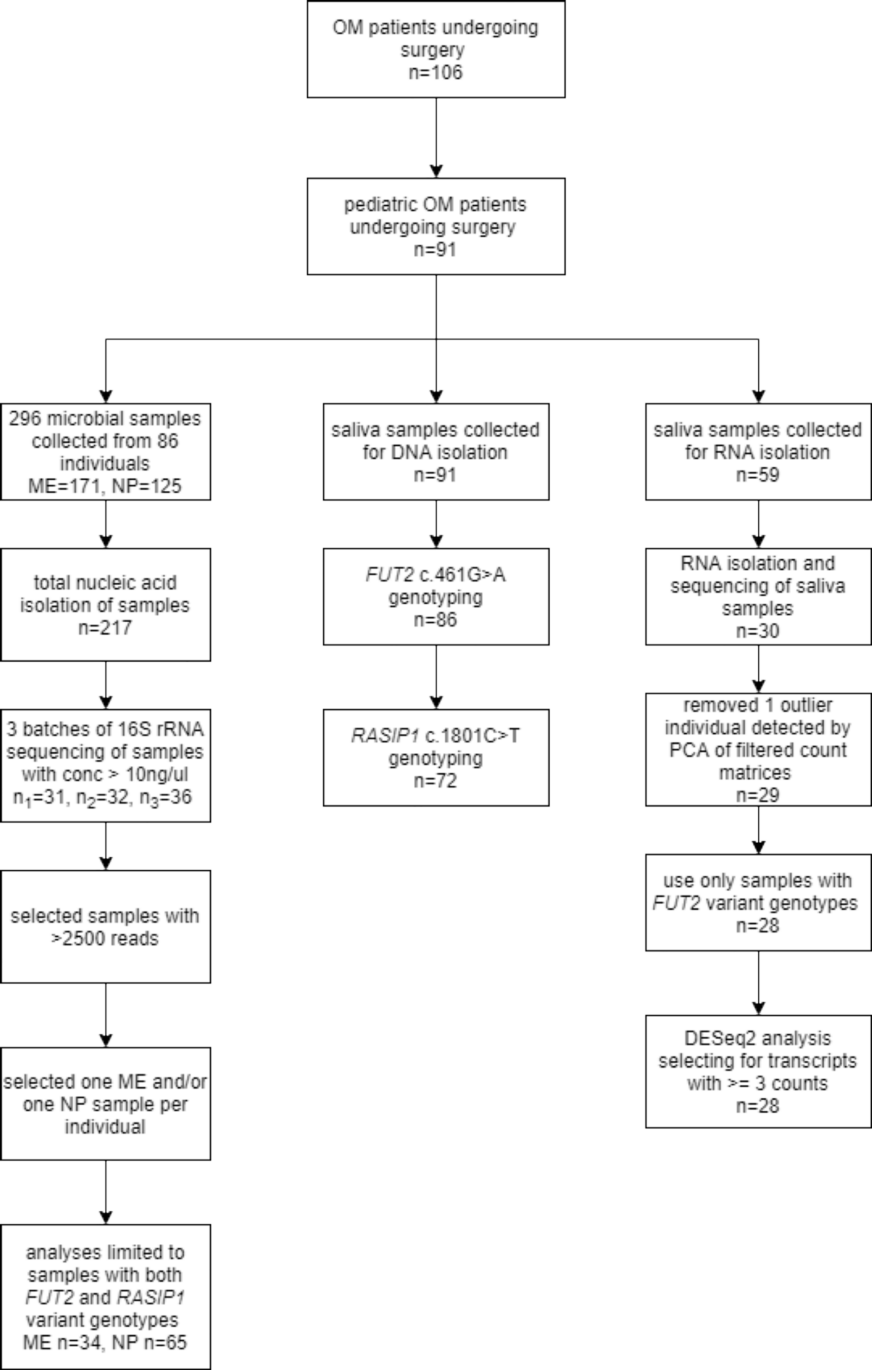
Study flowchart. The flowchart shows the number of saliva and microbial samples included for genotyping, RNA-seq and microbiota analyses.

Clinical data were obtained from 91 pediatric patients undergoing surgery for OM, with information on age, sex, self-reported ethnicity, family history, breastfeeding history, history of exposure to smoking, OM diagnoses and surgical technique (**Table 1**). We also had clinical information and samples from 15 adult patients with OM, but these samples were removed from further analyses because of marked differences in expression and microbiota profiles due to age (**Figure 1**). DNA samples were collected from the 91 pediatric patients with OM using the Oragene-DNA OGR-500 or OGR-575 kits (DNA Genotek, Ottawa, Ontario, Canada).

**Table 1:** Characteristics of OM patients by dataset.

Cohort characteristics^a^	Entire pediatric cohort (n=91)	Microbiota (n=65)	RNA-seq (n=28)
Sample type	Saliva, middle ear swab/aspirate/mucosa, nasopharynx swab	Middle ear swab/aspirate/mucosa, nasopharynx swab	Saliva
Median age (years)	2.0	2.0	2.3
% Female	33.0%	32.3%	17.9%
% Self-reported ethnicity	74.7% White, 11.0% Hispanic, 1.1% Asian, 12.1% other or mixed	80.0% White, 9.2% Hispanic, 1.5% Asian, 9.3% other or mixed	85.7% White, 10.7% Hispanic, 3.6% Asian
*FUT2* c.461G>A genotype	25.6% GG, 48.8% GA, 25.6% AA	21.5% GG, 52.3% GA, 26.2% AA	18.5% GG, 51.9% GA, 29.6% AA
Otitis media type			
- % Recurrent/acute^b^	74.7%	72.3%	78.6%
- % Chronic/effusive^b^	16.5%	12.3%	7.1%
- % Both/either	8.8%	15.4%	14.4%
Otitis media surgery			
- % Ventilation tubes	91.2%	93.8%	85.7%
- % Tympanoplasty	8.8%	6.2%	14.3%
% Breastfed	89.0%	89.2%	82.1%
% Smoking Exposure	13.2%	13.8%	25.0%
% (+) Family history	63.7%	49.2%	42.9%

a Statistical tests for effect of FUT2 variant on distribution in overall cohort—

Sex: Chi-squared test of independence p= 0.45.

Age: Wilcoxon Rank Sum p=0.02.

Ethnicity: Chi-squared test of independence (White vs non-White) p=0.63.

Hardy Weinberg Equilibrium p=0.94.

b Recurrent/acute OM (RAOM), defined as ≥3 OM episodes in 6 months or ≥4 OM episodes in 12 months; Chronic/effusive OM (COME), defined as ME effusion persisting for ≥2 months (Rosenfeld et al., 2016).

Saliva samples were also collected from pediatric patients with OM using Oragene-RNA RE-100 kits and sufficient RNA was isolated from 30 samples using the manufacturer’s protocol (**Figure 1**). A total of 296 microbial samples were obtained from the ME (n=171) and NP (n=125) of 86 individuals, including 74 ME swabs, 86 ME aspirates, and seven ME mucosal tissue samples. Four ME cholesteatoma/granuloma tissue samples and 125 NP swabs were also collected (**Figure 1**). Microbial DNA was isolated from 217 (73%) samples using the Epicentre Masterpure Complete DNA Purification Kit (Lucigen, Middleton, WI, USA); the rest of the samples from which no microbial DNA was isolated were excluded from further study (**Figure 1**).

### Human DNA Sequencing for *FUT2* and *RASIP1* Variants

A variant in *RASIP1* c.1801C>T (p.Arg601Cys; rs2287922) is in moderate LD with the *FUT2* variant c.461G>A (r^2 =^ 0.82) and with the rs681343 variant (r^2 =^ 0.65) that was associated with childhood ear infections (Pickrell et al., 2016; Buniello et al., 2019). Sanger sequencing was performed for the *FUT2* NM_000511.6:c.461G>A and *RASIP1* NM_017805.3:c.1801C>T variants using DNA from saliva samples of pediatric patients with OM. Both variants were in Hardy-Weinberg equilibrium within the entire cohort and in each cohort used for RNA-seq and microbiota analyses (**Table 1**).

### Human RNA-Sequencing and Analysis

Thirty salivary RNA samples (median RIN=7.1) were submitted for RNA-sequencing at the University of Colorado Denver Genomics and Microarray Core, as previously described (Larson et al., 2019). In summary, RNA samples were processed using the Nugen Trio RNA-Seq Kit (Tecan, Redwood City, CA, USA). Sequencing was performed on an Illumina HiSeq 4000 with an average of 31 million reads per sample. Reads were trimmed using the FASTX-Toolkit v0.0.13 and aligned using STAR v2.5.3a (Dobin et al., 2013). Principal components analysis (PCA) was performed on this dataset and one outlier sample was removed from further analyses due to not clustering with other samples (**Supplementary Figure 1**). Transcript counts were summarized at the gene level and analyses included genes with an average read count >3. DE analysis was performed on 28 samples (**Figure 1**) according to carriage of the *FUT2* c.461G>A variant using the DESeq2 package in R (Love et al., 2014), with correction for age, sex and batch effects (**Supplementary Figure 1**). Results were considered significant for genes with log_2_-transformed fold change > ± 2 and false discovery rate (FDR)-adjusted p-value <0.05 using the Benjamini-Hochberg method.

### Network and Pathway Analysis

*FUT2*, *RASIP1* and DE genes were used as input in NetworkAnalyst for construction of a protein-protein interaction network using the IMEx interactome database (Xia et al., 2014; Xia et al., 2015). Pathway enrichment analysis was performed on the resulting network using the KEGG and PANTHER GO-slim BP databases in NetworkAnalyst (Kanehisa and Goto, 2000; Kanehisa, 2019; Mi et al., 2019; Kanehisa et al., 2021). Pathways with an FDR-adjusted p<0.05 were deemed significantly enriched.

### 16S rRNA Sequencing and Microbiota Analysis

A total of 171 ME and 125 NP samples were obtained from 86 Coloradan pediatric patients with OM and submitted for 16S rRNA sequencing. Microbial DNA isolation was performed using the Epicentre MasterPure™ Kit. In order to test for contaminating bacterial DNA in reagents or plastics, every batch of samples that was submitted for 16S rRNA gene PCR and sequencing included ≥3 negative process controls. Bacterial profiles were determined by broad-range PCR amplification and sequence analysis of the 16S rRNA gene V1V2 regions, as previously described (Santos-Cortez et al., 2018; Frank et al., 2021). Illumina paired-end sequencing was performed on MiSeq using the 600 cycle version 3 kit. Assembled and quality-filtered sequences were aligned and classified with SINA (1.3.0-r23838) using the 418,497 bacterial sequences in Silva 115NR99 (Pruesse et al., 2012; Quast et al., 2013). Operational taxonomic units (OTUs) were produced by clustering sequences with identical taxonomic assignments (median: 115,176 sequences/sample; interquartile range: 46,274.5 – 170,300.0). Goods coverage scores were ≥99.7% for all samples, indicating adequate depth of sequence coverage for all samples. Of the 296 microbial samples submitted for sequencing, 79 did not pass quality control (DNA concentration ≥10 ng/ul; 2500 reads after sequencing; **Figure 1**). Because it was not possible to determine whether the lack of microbial DNA is due to a relatively sterile ME or from a sample collection issue, these 79 samples were excluded. Bacterial alpha-diversity indices (richness, diversity, and evenness; Robertson et al., 2013) were tested for association with carriage of each of the *FUT2* c.461G>A or *RASIP1* c.1801C>T variants independently *via* Wilcoxon test and adjusted for ethnicity (Robertson et al., 2013). Associations of individual OTUs with *FUT2* c.461G>A and *RASIP1* c.1801C>T variants were assessed using linear regression with sample batch as a covariate. To minimize multiple-comparisons, only taxa with a prevalence >10% and relative abundance >1% were included in the analysis. Beta-diversity was determined *via* PERMANOVA using the Morisita-Horn dissimilarity index and adjusted for age, sex and batch effects. R software was used for data analyses and figure generation.

### Gene Expression in Infected Murine Middle Ear

All animal experiments were performed according to the recommendations of the Guide for the Care and Use of Laboratory Animals of the National Institutes of Health and carried out in strict accordance with an approved Institutional Animal Care and Use Committee (IACUC) protocol (A13-022) of the Veteran Affairs Medical Center (San Diego, CA). All animal experiments employed the best efforts for minimizing animal suffering under general anesthesia according to the NIH guidelines.

For gene array studies, wild-type (WT) C57/WB F1 hybrid mice were purchased from the Jackson Laboratory (Bar Harbor, ME USA). NTHi strain 3655 (non-typeable, biotype II, originally isolated from the ME of a child with OM in St Louis, MO USA) was cultured in defined liquid media (Coleman et al., 2003). To induce ME infection, mice were deeply anesthetized with an intraperitoneal injection of rodent cocktail (13.3 mg/ml ketamine hydrochloride, 1.3 mg/ml xylazine, 0.25 mg/ml acepromazine; at 0.1-0.2 ml per 25-30 g body weight of the mouse). The bullae were bilaterally exposed through soft tissue dissection *via* a ventral approach. A hole was made in the bulla with a 23 gauge needle, allowing approximately 5 μl of NTHi inoculum (~5x10^4^ CFU/mL) to be injected using a Hamilton syringe with a 30-gauge needle. After the injection of NTHi inoculum, the tympanic membranes were visually inspected and confirmed to be intact. The incision was then stapled and the mice were given normal saline and analgesics (buprenorphine at 0.05mg/Kg) subcutaneously while recovering on the heated mat. Following recovery from anesthesia the mice appeared healthy, with a clinical activity index ≤ 3 throughout the duration of OM.

Gene array data were generated as previously described (Hernandez et al., 2015). In summary, forty mice per time point were inoculated bilaterally with NTHi. Mucosal tissue and exudate were harvested from 20 mice at each of the following intervals – 0 hours (0h, no treatment), 3h, 6h, 1 day (1d), 2d, 3d, 5d and 7d after inoculation – then pooled. The tissue was homogenized in TRIzol (Life Technologies, Carlsbad, CA) and total RNA extracted, reverse transcribed and re-transcribed *in vitro* to generate biotinylated cRNA probes that were hybridized to 2 Affymetrix MU430 2.0 microarrays. Hybridization intensity data were median-normalized and differences in gene transcript expression levels evaluated using variance-modeled posterior inference (VAMPIRE) (Hsiao et al., 2005). Bonferroni multiple testing correction (αBonf < 0.05) was applied to identify only those genes with the most robust changes. The data were duplicated at each time point to obtain a second, independent biological replicate. Thus each data point represents 2 separate samples consisting of 20 mice each, and 4 Affymetrix arrays. A total of 3,605 genes, approximately 14.4% of the mouse genome, defined the signature of acute, NTHi-induced OM across time. Hybridization of RNA to specific gene probes was assessed at individual time points by comparison to uninfected MEs, after Bonferroni correction for multiple tests, using Genespring GX 7.3 (Agilent Technologies, Santa Clara, CA).

For single-cell RNASeq, the same ME inoculation protocol was followed, except that C57BL/6J mice (Jackson Labs) were employed. Single-cell samples for RNA-sequencing were generated from the entire contents of the mouse ME (Ryan et al., 2020). For each of three independent samples, tissue was harvested from both ears of six young adult C57-BL6 mice 6 hours after inoculation of the ME with NTHi. Single-cell libraries were generated using the 10X Genomics (Pleasanton, CA, USA) Chromium Single Cell 3’ Reagent Kit V2. cDNA synthesis, barcoding, and library preparation were then carried out on a 10X Genomics Chromium Controller according to the manufacturers’ instructions. After validating quality of cDNA library, sequencing was performed on an Illumina HiSeq 2500 (Illumina, San Diego, CA USA). Reads were demultiplexed and aligned to the murine reference genome (mm10 with annotations from Ensembl, release 84). 10X Genomics Cellranger aggr and Seurat were used to generate PCA clustering (Satija et al., 2015). The expression of well-recognized marker genes identified 24 distinct cell types (Ryan et al., 2020). Linearized relative expression levels of each gene examined in this study were log-transformed from single-cell mRNA copy numbers, normalized, and scaled for each cell type. Data were visualized in 10X Genomics cLoupe, with UMI numbers expressed colorimetrically for each cell.

## Results

### Cohort Summary

Samples were collected from 91 pediatric patients with OM with ages ranging from 8.7 months to 14.9 years old (median 2.0 years; **Table 1**). Of these, 86 had sufficient DNA sample for Sanger sequencing of *FUT2* c.461G>A (**Figure 1**) and 83.3% are homozygous or heterozygous for the *FUT2* variant. Carriage of the *FUT2* variant was not associated with age, sex, ethnicity or OM diagnosis among children with OM (**Table 1**). In the entire cohort and in each subset analyses, males were predominant (≥81%), which is a known phenomenon for OM (Paradise et al., 1997).

### Differentially Expressed Genes in OM Patients With the *FUT2* c.461G>A Variant

**Figure 2:**
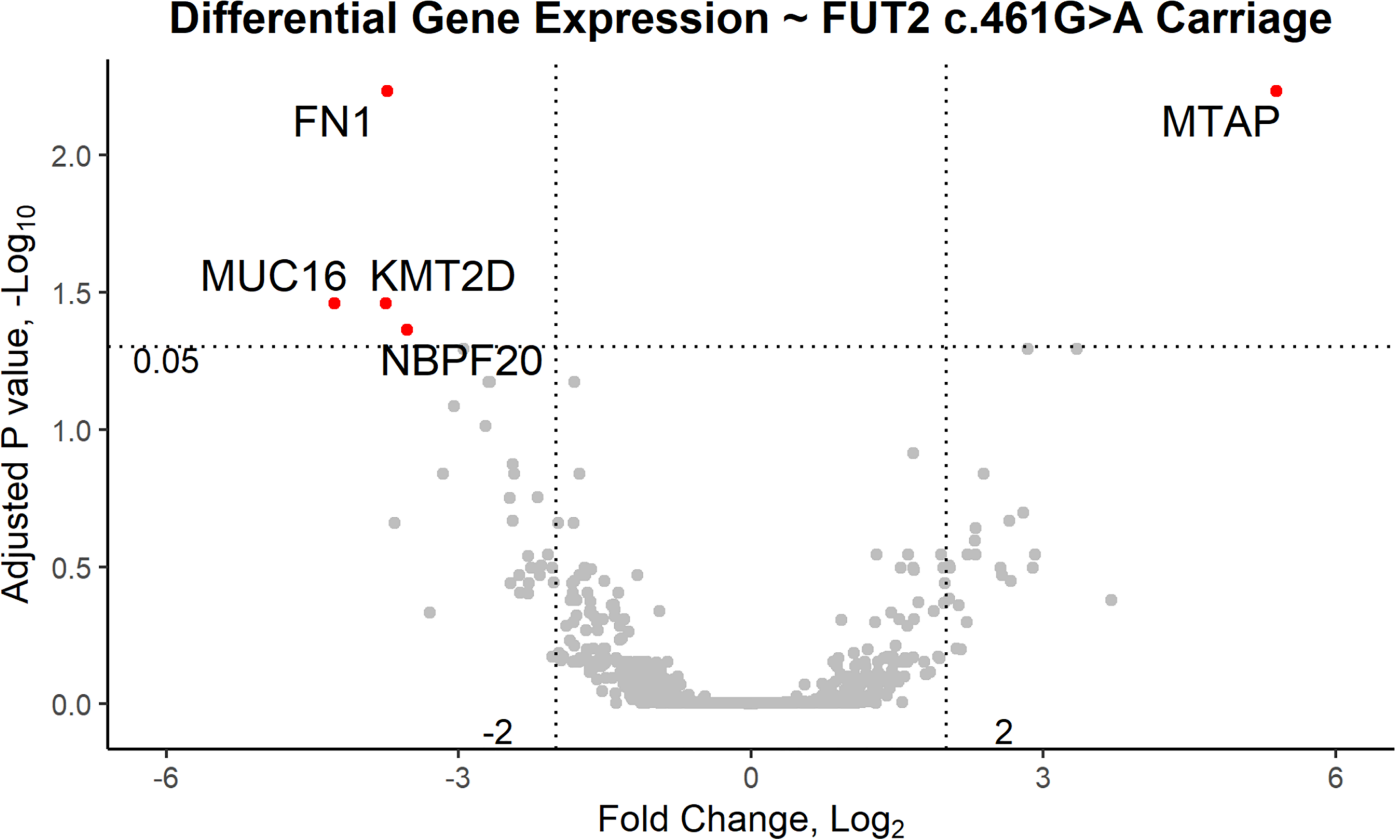
Volcano plot of differentially expressed genes based on carriage of the *FUT2* c.461G>A variant in patients with OM. In variant carriers, *KMT2D/MLL2, MUC16, NBPF20* and *FN1* were downregulated (FDR-adjusted p < 0.05, log2 fold change < -2) and *MTAP* was upregulated (FDR-adjusted p < 0.05, log2 fold change > 2).

RNA-seq data from 28 pediatric patients (0.8 to 14.8 years old; **Table 1**) passed QC and were available for analysis according to *FUT2* genotype. DE analysis was performed using *FUT2* c.461G>A variant carriage as the classifier (5 wildtype and 23 variant carriers) and with adjustment for age, sex and batch effects (**Table 1** and **Supplementary Figure 1**). Five DE genes were significant, namely: *FN1* (log-fold change = -3.7, FDR-adj-*p*=0.006); *KMT2D/MLL2* (log-fold change = -3.8, FDR-adj-*p*=0.04); *MUC16* (log-fold change = -4.3, FDR-adj-*p=*0.04); *MTAP* (log-fold change = +5.4, FDR-adj-*p=*0.006); and *NBPF20* (log-fold change = -3.5, FDR-adj-*p*=0.04). In carriers of the *FUT2* c.461G>A variant, *FN1*, *KMT2D/MLL2*, *MUC16* and *NBPF20* were downregulated whereas *MTAP* was upregulated (**Figure 2**).

**Figure 3:**
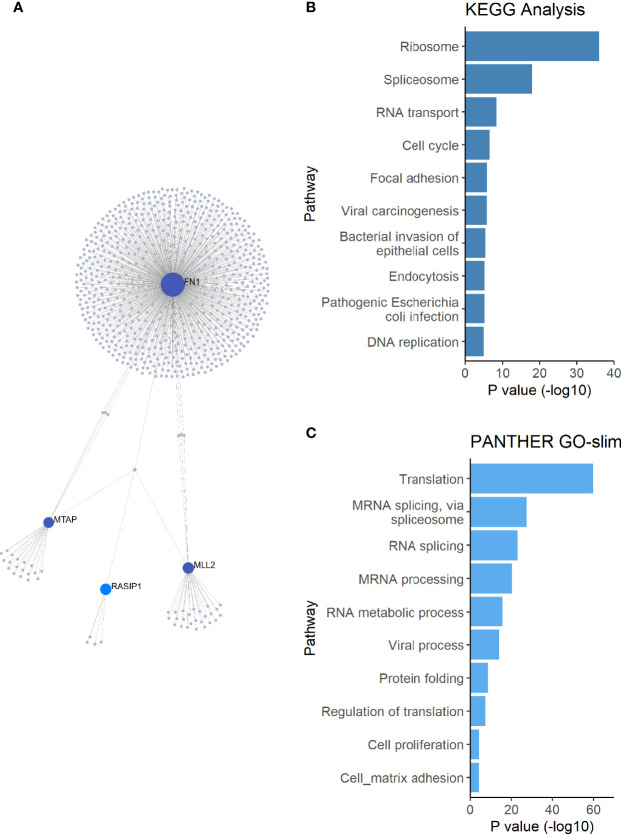
Network and pathway enrichment analysis of differentially expressed genes. **(A)** A single PPI network was constructed using the *FUT2, RASIP1* and the DE genes as input. **(B)** KEGG and **(C)** PANTHER GO-slim:BP pathway enrichment analysis results, showing the top 10 pathways with the smallest *p-*values. *MUC16* and *FUT2* are not connected to this network, suggesting a different mechanism for the interaction of these two genes in relation to OM.

To further investigate how *FUT2, FN1*, *KMT2D/MLL2*, *MUC16, MTAP*, *NBPF20* and *RASIP1* are related, these genes were used as input for network analysis. *RASIP1, FN1*, *KMT2D/MLL2* and *MTAP* were connected in a single protein-protein interaction network (**Figure 3A**). Pathway enrichment analysis of this network revealed 27 significant pathways in KEGG and 21 significant processes in PANTHER GO-slim BP, many of which overlap (**Figures 3B, C** and **Table 2**). Among these are processes pertaining to viral and bacterial infection, cell cycle regulation, apoptosis, and endocytosis (**Table 2**).

**Table 2:** Significant pathways within network connecting DE genes.

KEGG		PantherBP : GO-slim	
Pathway	FDR-adj-p	Pathway	FDR-adj-p
**Ribosome**	2.82E-34	**Translation**	2.37E-58
**Spliceosome**	1.60E-16	**MRNA splicing, *via* spliceosome**	3.15E-26
**RNA transport**	4.05E-07	**RNA splicing**	6.20E-22
**Cell cycle**	2.09E-05	MRNA processing	2.46E-19
**Focal adhesion**	8.29E-05	**RNA metabolic process**	7.10E-15
Viral carcinogenesis	8.66E-05	**Viral process**	3.14E-13
Bacterial invasion of epithelial cells	0.0002	Protein folding	5.78E-08
**Endocytosis**	0.0002	**Regulation of translation**	1.14E-06
Pathogenic E. coli infection	0.0002	**Cell proliferation**	0.001
**DNA replication**	0.0003	**Cell_matrix adhesion**	0.001
Proteoglycans in cancer	0.001	**Rhythmic process**	0.001
Huntington’s disease	0.001	**Negative regulation of apoptotic process**	0.002
Proteasome	0.002	MRNA 3’_end processing	0.002
**Regulation of actin cytoskeleton**	0.002	**Intracellular protein transport**	0.003
Carbon metabolism	0.003	**Vesicle_mediated transport**	0.003
Adherens junction	0.004	**Glycolytic process**	0.005
Endocrine and other factor-regulated calcium reabsorption	0.004	**DNA replication**	0.01
mRNA surveillance pathway	0.004	RNA splicing *via* transesterification reactions	0.02
**Aminoacyl-tRNA biosynthesis**	0.004	**Receptor_mediated endocytosis**	0.02
Estrogen signaling pathway	0.008	Protein transport	0.02
Leukocyte transendothelial migration	0.01	DNA recombination	0.03
**Glycolysis/Gluconeogenesis**	0.02		
Hepatitis B	0.03		
Shigellosis	0.04		
Pyruvate metabolism	0.04		
Salmonella infection	0.04		
Bladder cancer	0.049		

Overlap between databases in bold.

### Differentially Expressed Genes Were Also Significantly Regulated in Infected ME of Wildtype Mice

**Figure 4:**
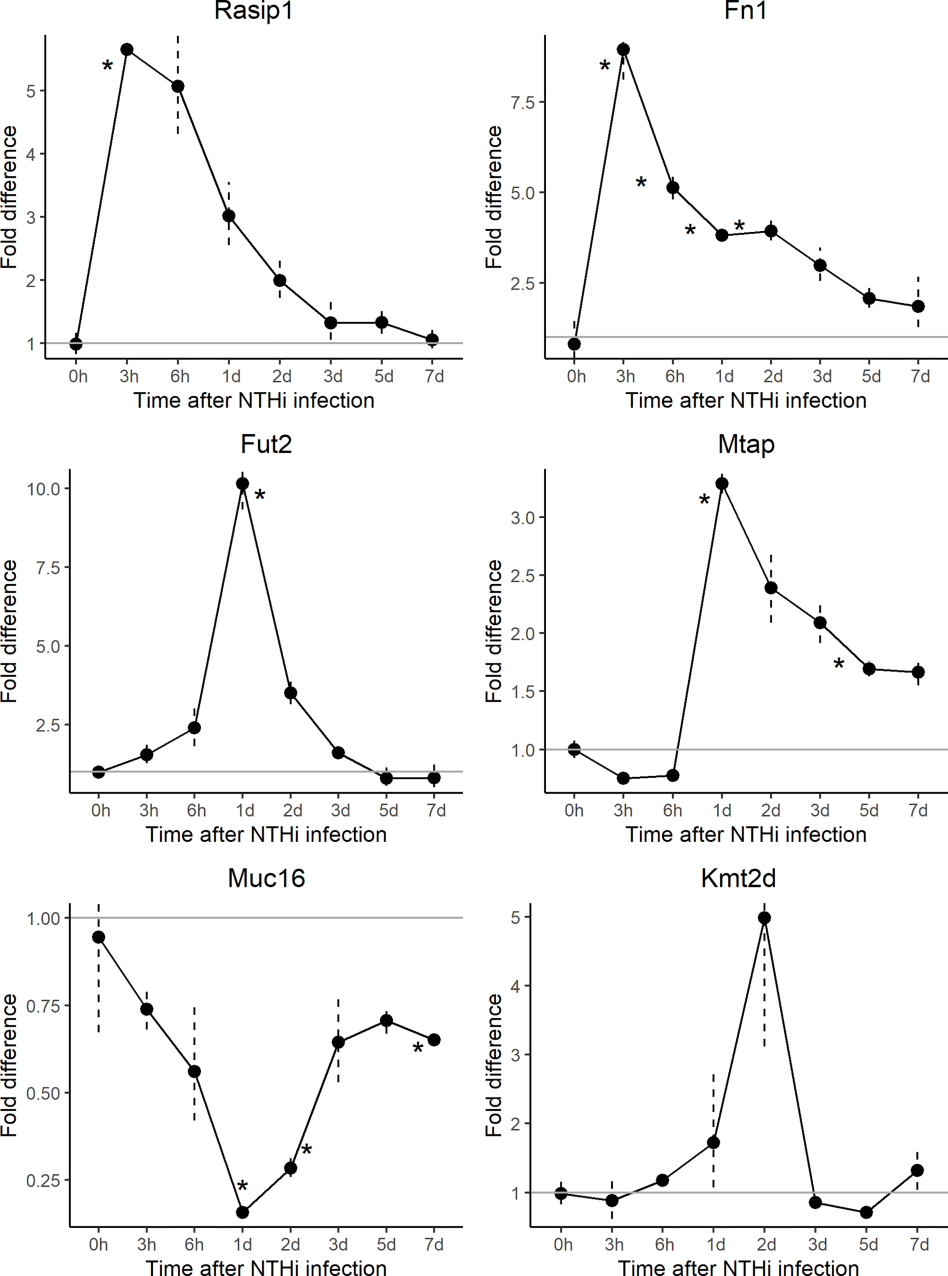
Gene array expression data for select genes post-inoculation with non-typeable *Haemophilus influenzae (NTHi).* Mouse middle ear expression of select genes across different time points, shown as fold change in middle ears inoculated with NTHi as compared to placebo. *Fut2*, *Muc16* and *Mtap* reached peak change in expression at 24 hours post-inoculation while *Muc16* demonstrated sustained downregulation. On the other hand, *Rasip1* and *Fn1* reached peak upregulation at 3 hours post-inoculation. In this experiment, time point 0h represents uninfected middle ear. *p < 0.05; see **Table 3** for gene expression values by time point and gene.

To further understand the role and interactions between *FUT2, RASIP1* and DE genes, expression of orthologs *Fut2, Fn1*, *Kmt2d*, *Muc16, Mtap* and *Rasip1* were measured by gene array in ME of wildtype mice at multiple time points (from 3 hours to 7 days) post-infection with NTHi (**Figure 4** and **Tables 3**, **4**). The *NBPF* gene family results from segmental duplication events in primate, thus an ortholog for *NBPF20* is not present in mice (Vandepoele et al., 2005). Expression of *Fut2, Rasip1* and *Mtap* were significantly increased after inoculation, with *Fut2* and *Mtap* peaking around one day post-inoculation, and *Rasip1* and *Fn1* at 3 hours post-inoculation (**Figure 4**). Additionally, expression of *Muc16* was significantly decreased one day post-inoculation. For *Fn1, Mtap* and *Muc16*, DE was sustained through days 2-7 post-inoculation, including when OM is supposedly in recovery phase (Hernandez et al., 2015). *Kmt2d* showed no significant changes in ME expression at any point during the 7 days when compared to control mice (**Figure 4**).

**Table 3:** Mouse ME gene expression values by time point.

Gene	Probe	Time	Fold diff.	lower	upper	p-value (*<0.05)
*Fut2*	143862_at	0h	0.99	0.86	1.14	0.96
		3h	1.53	1.27	1.85	0.27
		6h	2.39	1.81	3.16	0.20
		1d	10.14	9.32	11.03	0.02*
		2d	3.49	3.13	3.89	0.55
		3d	1.60	1.48	1.74	0.11
		5d	0.79	0.55	1.13	0.63
		7d	0.80	0.51	1.28	0.72
*Rasip1*	1428016_at	0h	0.99	0.83	1.17	0.95
		3h	5.65	5.55	5.74	0.006*
		6h	5.07	4.32	5.95	0.06
		1d	3.01	2.56	3.55	0.09
		2d	1.99	1.72	2.31	0.13
		3d	1.33	1.06	1.66	0.43
		5d	1.33	1.15	1.54	0.30
		7d	1.06	0.92	1.21	0.76
*Fn1*	1437218_at	0h	0.80	0.40	1.60	0.80
		3h	8.94	8.11	9.85	0.03*
		6h	5.13	4.80	5.48	0.03*
		1d	3.82	3.70	3.94	0.01*
		2d	3.93	3.67	4.21	0.03*
		3d	2.97	2.55	3.47	0.09
		5d	2.07	1.81	2.35	0.11
		7d	1.85	1.28	2.67	0.34
*Mtap*	1451345_at	0h	0.10	0.93	1.08	0.98
		3h	0.75	0.73	0.78	0.08
		6h	0.78	0.73	0.83	0.15
		1d	3.29	3.20	3.37	0.01*
		2d	2.39	2.09	2.73	0.10
		3d	2.09	1.92	2.28	0.07
		5d	1.69	1.63	1.76	0.047*
		7d	1.67	1.55	1.79	0.09
*Muc16*	1432358_at	0h	0.95	0.67	1.33	0.90
		3h	0.74	0.68	0.80	0.17
		6h	0.56	0.42	0.75	0.30
		1d	0.16	0.15	0.16	0.01*
		2d	0.28	0.26	0.31	0.047*
		3d	0.64	0.53	0.78	0.27
		5d	0.71	0.67	0.75	0.10
		7d	0.65	0.64	0.67	0.04*
*Kmt2d*	1427555_at	0h	0.99	0.83	1.17	0.95
		3h	0.88	0.62	1.26	0.79
		6h	1.18	1.09	1.27	0.28
		1d	1.73	1.08	2.77	0.46
		2d	4.98	3.12	7.97	0.18
		3d	0.85	0.80	0.91	0.25
		5d	0.71	0.62	0.81	0.24
		7d	1.32	1.04	1.67	0.45

*Denotes p-values < 0.05.

**Table 4:** Comparison of DE gene regulation in human saliva of *FUT2* c.461G>A variant carriers vs non-carriers and NTHi- vs placebo-inoculated mouse middle ear (ME).

Gene	FUT2 Variant Carrier vs Wildtype (human saliva expression)	NTHi- vs PBS-inoculated (mouse ortholog ME expression)
*FUT2*	*Genotype as classifier variable*	Upregulated in NTHi at 1 day
*RASIP1*	*Genotype as classifier variable*	Upregulated in NTHi at 3 hours
*FN1*	Downregulated in variant carriers	Upregulated in NTHi, peak at 3 hours
*MTAP*	Upregulated in variant carriers	Upregulated in NTHi, peak at 1 day
*MUC16*	Downregulated in variant carriers	Downregulated in NTHi, peak at 1 day
*KMT2D/MLL2*	Downregulated in variant carriers	Not significant
*NBPF20*	Downregulated in variant carriers	*Not applicable*

**Figure 5:**
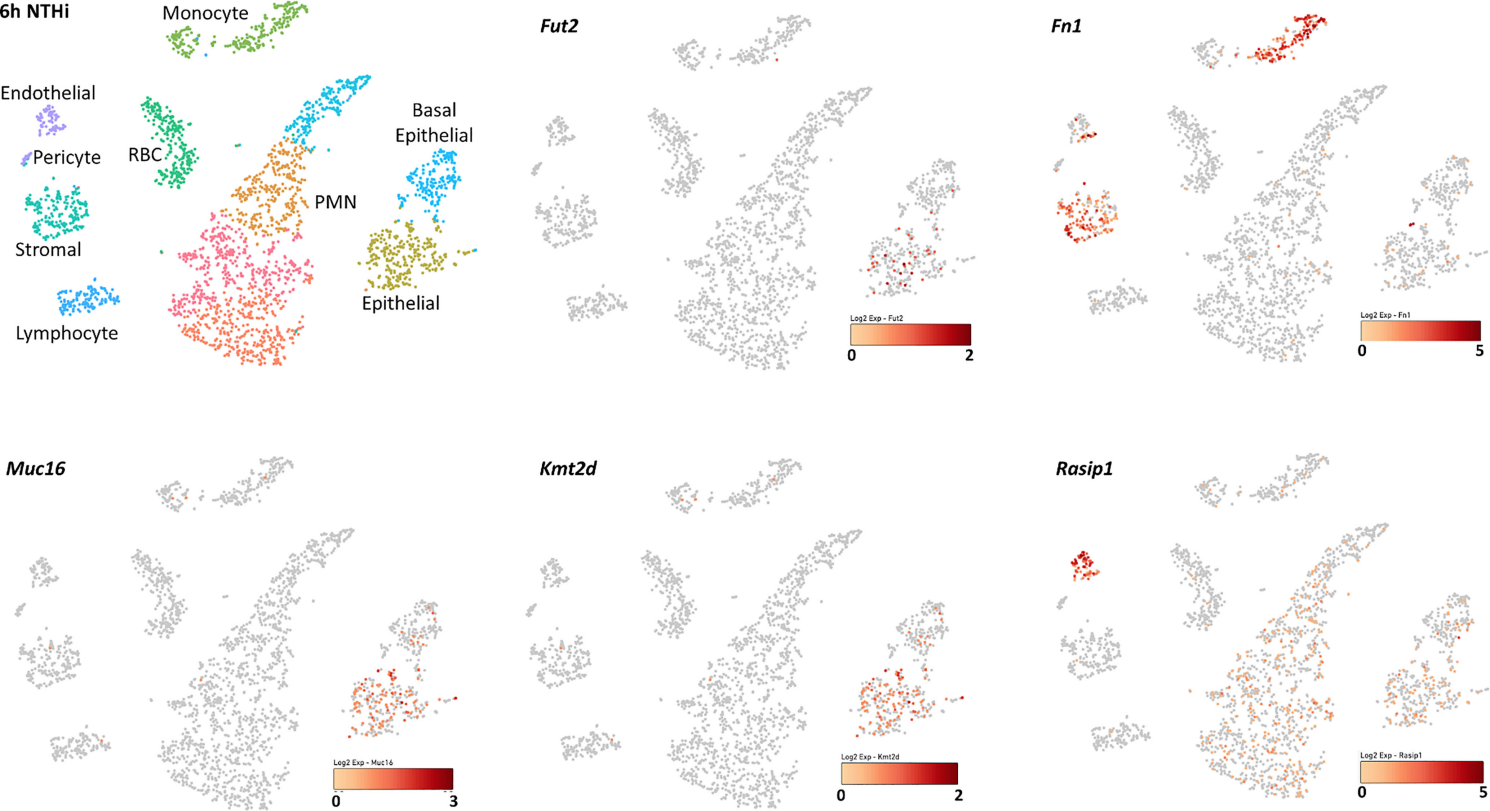
Single-cell RNA-seq expression data from mouse middle ear 6 hours after inoculation with NTHi. Expression of *Fut2, Fn1, Muc16, Kmt2d* and *Rasip1* in wildtype mouse middle ear, 6 hours post-infection. Cell types of the various PCA clusters were identified by the expression of unique marker genes. Darkness of color (*red* for each gene) indicates level of UMI expression by each cell, according to the associated log_2_ scale. Epithelial cell clusters were identified based on the expression of *Krt18* and/or *Krt19*. Basal epithelial cells also express *Krt14*, while ciliated epithelial cells express *Hydin*. Stromal cells are identified by *Col1a2*. Vascular endothelial cells express *Egfl7* and *Flt4*, lymphatic endothelial cells *Egfl7* and *Flt1*. Melanocytes express *Mlana* and pericytes *Rgs5*. Monocytes express *Csf1r*, lymphocytes *Ptprcap*, polymorphonuclear cells *Il1f9* and *Stfa2l1*, and red blood cells *Hba-a1*.

Single-cell RNA-sequence (scRNA-Seq) data were derived from the MEs of NTHi-infected mice six hours after inoculation (**Figure 5** and **Table 5**). In uninfected ME (time point 0h), *Fut2* was expressed primarily in ciliated epithelial cells (*Hydin*+). *Muc16* was expressed in most epithelial cells except basal epithelial cells (*Krt14*+). *Rasip1* was expressed in most endothelial cells (*Egfl7*+) and *Fn1* mostly in stromal cells (*Col1a2*+) and melanocytes (*Mlana*+). *Mtap* and *Kmt2d* were modestly expressed in all ME cell types. Six hours after ME inoculation with NTHi, when overall expression data was strongest (**Figure 5**), *Fut2* had increased expression in non-ciliated epithelial cells (*Krt18/19*+) and *Muc16* in all epithelial cell types. *Rasip1* continued to be expressed in endothelial cells, but was also observed in polymorphonuclear cells (PMNs) and monocytes (*Csf1r*+). *Fn1* increased expression in stromal cells and monocytes and some endothelial cells (**Figure 5**). *Mtap* and *Kmt2d* remained moderately expressed in all ME cell types except infiltrating PMNs and red blood cells. Level of gene expression per cell peaked at 1 day, and then declined (**Table 5**). Taken together, the mouse ME expression profiles for *Fut2, Rasip1* and DE genes support the findings of DE genes in OM patients using RNA-seq data from saliva (**Table 4**), and also the overall expression of these genes in other human mucosal tissues (**Table 6**).

**Table 5:** Single-cell RNA-seq expression levels in mouse ME by time point after NTHi inoculation.

Gene	0 hour	6 hours	1 day	5 days	7 days
*Fut2*	modest in ~10% of ciliated epithelial cell and a few other non-basal epithelial cells	modest in ~10% of non-ciliated non-basal epithelial cells	moderate in ~30% of epithelial cells	modest in only a few epithelial cells	modest in ~10% of ciliated and other non-basal epithelial cells
			~10% of vascular endothelial cells and a few PMNs		
*Rasip1*	moderate in most endothelial cells, both vascular and lymphatic	very strong in most endothelial cells, modest in ~20% of PMNs and ~5% of monocytes	strong in vascular endothelial cells,	strong in most vascular endothelial, modest in ~50% of other cells but stromal, lymphocytes	moderate in most endothelial cells
			modest in some PMNs, monocytes		
*Fn1*	strong in ~50% of stromal cells, melanocytes, a few endothelial cells and monocytes	strong in most stromal cells, moderate in most monocytes, a few endothelial cells	very strong in most monocytes, some stromal cells, and some vascular endothelial cells	very strong in all stromal cells; moderate in ~50% of monocytes and vascular endothelial cells	strong in all vascular endothelial cells, moderate in most stromal cells, modest in ~10% of monocytes
*Mtap*	modest in ~10% of all cell types	modest in ~10% of all cell types but PMNs, RBCs	moderate in most vascular epithelial cells, ~50% of stromal cells and epithelial cells, some monocytes	modest in ~20% of all cell types but PMNs, RBCs	modest in 10-20% of all cell types but ciliated epithelial cells, RBCs
*Muc16*	moderate in most epithelial cells, excluding basal cells	modest in most non-basal epithelial cells	moderate in most non-basal epithelial cells	moderate in non-basal epithelial cells, very modest in ~10% of basal epithelial cells	moderate in most non-basal epithelial cells, modest in ~10% of basal epithelial cells
*Kmt2d*	modest in ~10-20% of all cell types	modest in ~10-20% of all cell types except PMNs, RBCs	moderate in most vascular endothelial and ~50% of epithelial cells; modest in most stromal cells, monocytes, PMNs	modest in 50% of epithelial cells and ~10-20% of all other cell types but RBCs	modest in ~10-20% of all cell types but RBCs

Very modest expression = <0.5 x log2 UMI (transcript)/cell.

Modest expression = 0.5-1 x log2 UMI/cell.

Moderate expression = 1.5-2 x log2 UMI/cell.

Strong expression = 2.5-3 x log2 UMI/cell.

Very strong expression = 3.5-5 x log2 UMI/cell.

PMNs, polymorphonuclear cells; RBCs, red blood cells.

**Table 6:** Known RNA and protein expression profiles of *FUT2*, *RASIP1* and DE genes in human tissues.

Gene	RNA Expression (GTEx Consortium; Lonsdale et al., 2013)	Protein Expression (Human Protein Atlas; Uhlen et al., 2015)
*FUT2*	Minor salivary gland, esophagus-mucosa, small intestine-terminal ileum, colon-transverse, stomach, vagina	Medium expression in most organs/tissues including nasopharynx, lung and oral mucosa
*RASIP1*	Lung, adipose-visceral (omentum), breast-mammary tissue, adipose-subcutaneous, spleen, uterus	Medium expression in gallbladder, kidney, placenta, smooth muscle; low expression in adrenal gland, salivary gland, epididymis, appendix, tonsil, cerebral cortex, colon
*FN1*	Cultured fibroblasts, artery-aorta, coronary, tibial	High expression in kidney; medium or low expression in many organs/tissues including low expression in nasopharynx, lung and oral mucosa
*KMT2D/MLL2*	Expression detected across all tissues/organs; highest in uterus, thyroid, brain-cerebellum	High expression in cerebral cortex, cerebellum, testis, and epididymis; medium or low expression in many organs/tissues including low expression in nasopharynx and lung
*MTAP*	Highest expression in cells-cultured fibroblasts, nerve-tibial, ovary, uterus	*Unavailable*
*MUC16*	Minor salivary gland, adipose-visceral (omentum), fallopian tube, testis, lung, cervix-endocervix	High expression in bronchus, fallopian tube, endometrium, uterine cervix; medium expression in salivary gland; low expression in nasopharynx

### ME and NP Microbiota Profiles of Patients Carrying the *FUT2* c.461G>A Variant

A total of 296 microbial samples were collected from the NP and ME of 86 children (**Figure 1**). For microbiota analyses, samples were filtered for: (1) those with >2500 16S rRNA sequencing reads; (2) one ME and one NP sample per individual where bilateral samples were collected (if bilateral, right-sided sample was used); and (3) available genotypes for *FUT2* and *RASIP1* variants (**Figure 1**). No differences were identified between right and left NP or ME samples from the same individuals in PCA and PERMANOVA analyses (data not shown). After filtering, 16S rRNA sequence data from 34 ME and 65 NP samples were analyzed according to carriage of the *FUT2* c.461G>A (p.Trp154*) variant.

**Figure 6:**
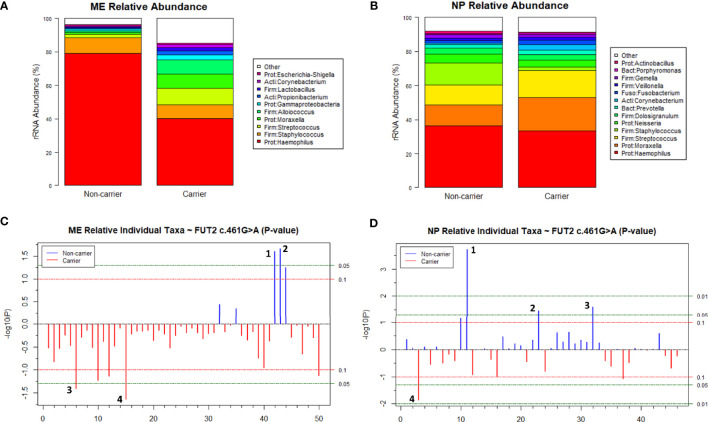
Relative abundance of individual taxa in middle ears (ME) and nasopharynges (NP) of carriers and non-carriers of the *FUT2* c.461G>A variant. **(A)** Cumulative relative abundance profiles in the ME of wildtype (n=8) and carriers (n=26) of *FUT2* c.461G>A. **(B)** Cumulative relative abundance profiles in the NP of wildtype (n=14) and carriers (n=51) of *FUT2* c.461G>A. Plots showing *p-*values for relative abundance of individual bacterial taxa in the **(C)** ME and **(D)** NP of wildtype versus variant carriers after adjusting for batch. *Blue lines* indicate taxa that were increased in wildtype, *red lines* for carriers. *Dashed lines* indicate significance thresholds where the *red line* is unadjusted-*p*=0.1 (non-significant) and *green lines* indicate unadjusted-*p*=0.05 and unadjusted-*p*=0.01. **(C)** In the ME, *Haemophilus* (1) and *Moraxella* (2) were nominally associated with wildtype, whereas *Propionibacterium* (3) and *Anoxybacillus* (4) were nominally associated with variant carriage. **(D)** In the NP, Candidate Division TM7 (1) was significantly associated with wildtype (FDR-adj-*p*=0.009). Additionally, *Selenomonas* (2) and *Actinobacillus* (3) were nominally associated with wildtype whereas *Propionibacterium* (4) was nominally associated with variant carriage.

In the ME, based on carriage of the *FUT2* variant, Chao1 which denotes bacterial richness was significant when all ethnic groups were included (*p*=0.03); however, all alpha-diversity indices were not significant when only individuals of European descent were included in analyses (**Supplementary Table 1**). Overall microbiota composition (i.e., beta-diversity) did not differ significantly by *FUT2* variant according to PERMANOVA analysis with adjustment for age, sex, or batch effects (**Figure 6A**). Additionally, the relative abundances of *Haemophilus* (nominal *p*=0.03) and *Moraxella* (nominal *p*=0.02) were increased with wildtype *FUT2* genotype, whereas increased *Propionibacterium* (nominal *p*=0.04) and *Anoxybacillus* (nominal *p*=0.02) were associated with presence (homozygous or heterozygous genotypes combined) of the variant (**Figure 6C** and **Supplementary Table 2**). Performing these analyses by genotype had no overall effect on results (**Supplementary Figure 2**).

In the NP, there were also no significant differences in alpha- or beta-diversity (**Supplementary Table 1** and **Figure 6B**). Similar to ME, *Propionibacterium* had increased relative abundance in the NP (nominal *p*=0.01) among carriers of the *FUT2* variant. In addition, the relative abundances of *Actinobacillus* (nominal *p*=0.03), *Selenomonas* (nominal *p*=0.03) and Candidate Division TM7 (*Saccharibacteria*; nominal *p*=0.0002) were increased in wildtype individuals (**Figure 6D**, **Supplementary Table 2**). When individual taxa were tested for association by genotype, no taxa were significant (**Supplementary Figure 2**). Note however that these *FUT2-*microbiota associations were nominal and were non-significant after FDR correction, with the exception of Candidate Division TM7 in the NP (FDR-adjusted *p*=0.009).

### RASIP1

**Figure 7:**
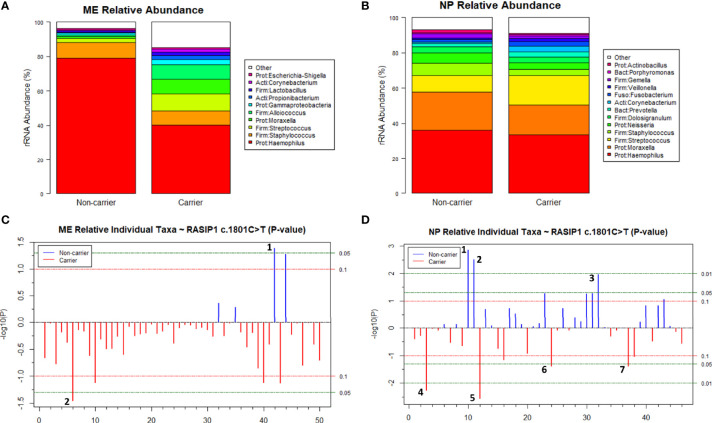
Relative abundance of individual taxa in the middle ears (ME) and nasopharynges (NP) of carriers and non-carriers of the *RASIP1* c.1801C>T variant. **(A)** Cumulative relative abundance profiles in the ME of wildtype (n=8) and carriers (n=26) of *RASIP1* c.1801C>T. **(B)** Cumulative relative abundance profiles in the NP of wildtype (n=15) and carriers (n=50) of *RASIP1* c.1801C>T. Plots showing *p-*values for relative abundance of individual bacterial taxa in the **(C)** ME and **(D)** NP of wildtype versus carriers after adjusting for batch. *Blue lines* indicate taxa that were increased in wildtype, *red lines* for carriers. *Dashed lines* indicate significance thresholds where the *red line* is unadjusted-*p*=0.1 (non-significant) and *green lines* indicate unadjusted-*p*=0.05 and unadjusted-*p*=0.01. **(C)** In the ME, *Gammaproteobacteria* (1) was nominally associated with wildtype, whereas *Propionibacterium* (2) was nominally associated with variant carriage. **(D)** In the NP, Candidate Division SR1 (1) and Candidate Division TM7 (2) were significantly associated with wildtype, and Chloroplast (5) with variant carriage (FDR-adj-*p*=0.05). Additionally, *Actinobacillus* (3) was nominally associated with wildtype, whereas *Propionibacterium* (4), *Staphylococcus* (6) and *Escherichia-Shigella* (7) were nominally associated with variant carriage.

Sanger sequencing of DNA samples confirmed that the *RASIP1* c.1801C>T and *FUT2* c.461G>A variants are in moderate LD in our cohort as the genotypes for 57 of 71 (80.3%) individuals were identical. In the ME, similar to findings with the *FUT2* variant, an increased relative abundance of *Haemophilus* (nominal *p*=0.04) was associated with wildtype genotype whereas increased *Propionibacterium* (nominal *p=*0.04) was associated with the *RASIP1* variant (**Figure 7C** and **Supplementary Table 3**). When analyzed by genotype, *Haemophilus* remained nominally associated with wildtype (**Supplementary Figure 3**). In the NP, increased abundance of *Propionibacterium* (nominal p=0.006), chloroplast (FDR-adjusted *p*=0.05), *Escherichia-Shigella* (nominal *p*=0.04) and *Staphylococcus* (nominal p=0.04) was associated with carriage of the *RASIP1* variant, whereas increased abundance of Candidate Division SR1 (FDR-adjusted *p*=0.05), Candidate Division TM7 (FDR-adjusted *p*=0.05), and *Actinobacillus* (nominal p=0.01) was associated with wildtype genotype (**Figure 7D** and **Supplementary Table 3**).

## Discussion

Variants in *FUT2*, including the c.461G>A (p.Trp154*) variant investigated here, have been associated with increased susceptibility to OM but their functional role in OM pathology has not been fully elucidated. Although *FUT2* has been studied by many groups, to our knowledge this is the first study in which RNA-seq data combined with ME and NP microbiotas have been examined in relation to carriage of the *FUT2* c.461G>A variant. Our results suggest that the *FUT2* variant confers OM susceptibility through its modulation of *MUC16* expression and downstream induction of *FN1* and *MTAP* after microbe binding and pathogen colonization (**Figures 2**, **3** and **Table 2**). These DE findings were supported by similar regulation of expression in NTHi-infected ME of wildtype mice, whether by bulk mRNA-seq or single-cell RNA-seq data (**Figure 4** and **Tables 3**, **4**). Because these genes were differentially regulated in response to OM in the infected wildtype mouse ME, the results of this DE analysis suggest that the *FUT2* c.461G>A variant magnifies the downstream response to infection (for example, downregulated *MUC16*, upregulated *MTAP*), and/or reverses the direction of regulation (e.g. downregulation of *FN1* in carriers of the *FUT2* variant; **Figures 2**, **4** and **Table 4**). Alternatively, DE genes may vary depending on the predominant otopathogen during infection: in other words, whether commensal or otopathogenic bacteria bind to ME mucosal epithelium *via* A antigen, the expression of which is affected by heterozygous or homozygous genotype for the *FUT2* c.461G>A variant (**Figures 6**, **7**; Santos-Cortez et al., 2018).

*RASIP1* is expressed in ME endothelial cells and provides another avenue for investigation in relation to *FUT2* c.461G>A variant carriage. *RASIP1* c.1801G>T, previously identified by GWAS to be in LD with *FUT2* c.461G>A (Pickrell et al., 2016), is also in moderate LD with *FUT2* c.461G>A in the sample set. *RASIP1* is part of the PPI immune network including *MTAP, KMT2D* and *FN1* (**Figure 3A** and **Table 2**), which led us to question whether the expression and microbiota effects we observed were being driven by the *RASIP1* missense variant rather than the *FUT2* stop variant. When examining the changes in the expression of these genes in wildtype mice after NTHi inoculation, *Rasip1* and *Fn1* expression peaked at 3 hours post-inoculation, whereas *Fut2* peaked at one day post-inoculation, in concordance with *Muc16* and *Mtap* expression (**Figure 4** and **Table 3**). Additionally, in the single-cell RNA-seq data from mouse ME, we observed *Rasip1* and *Fn1* expression in endothelial cells versus epithelial expression of *Fut2* and *Muc16* (**Figure 5** and **Table 5**). When examined together, these expression profiles strongly support *FUT2* as mediating OM susceptibility within the ME mucosal epithelium. In particular, the downregulation of *MUC16* in OM patients with the *FUT2* stop variant might indicate a prolonged recovery phase when *MUC16* is expected to return to normal levels as part of the normal response to acute OM. *MUC16* downregulation is therefore a potential avenue for future research, for example, whether this effect of *FUT2* knockdown is a mechanism for an acute infection to proceed to recurrence or chronicity (Kerschner et al., 2013).

Dysbiosis of the NP and ME mucosal microbiotas is supported by our data here and in our previous studies in which the ME of *FUT2* c.461G>A variant carriers were enriched in potentially otopathogenic taxa such as *Propionibacterium*, and decreased for established otopathogens *Haemophilus* and *Moraxella*, although these associations were nominal (Santos-Cortez et al., 2018). This could be attributed to the effect of *FUT2* c.461G>A on pathogen binding, wherein those homozygous for the *FUT2* variant are non-secretors of ABO(H) antigens on the epithelia surface (**Table 6**); these antigens can serve as ligands to which some bacteria may bind and thus affect the commensal and pathogen loads of the NP and ME. Interestingly the only bacterial taxon that has a significant association with the *FUT2* variant after correction for multiple testing is Candidate Division TM7, which is also known as *Saccharibacteria* (**Figure 6**). Little is known about *Saccharibacteria* and its reported associations with human mucosal disease have been variable, though there is some evidence that it parasitizes other bacteria and can kill its host bacterium, thereby modulating the overall microbiota (Bor et al., 2019).

The change in relative abundance of chloroplast in the NP corresponding to *RASIP1* variant carriage is an unusual result. This is potentially due to a sequence misclassification of cyanobacteria in the reference database rather than systematic contamination during isolation from the kit or reagents. Though general contamination is a possible explanation, if this were the case its presence would be detected among all samples or the effect would be eliminated by the adjustment for batch during analyses. Furthermore, chloroplast contamination would be negatively correlated with number of reads per sample as contamination would be less prominent in samples with higher bacterial loads. However, we did not observe these in our samples and during analyses. Thus, it is unlikely that the identification of chloroplast as being differentially abundant in carriers of the *RASIP1* variant is due to general contamination, though random, non-systematic contamination cannot be ruled out. Note that the main findings in this work are more likely explained by carriage of the *FUT2* variant and not the *RASIP1* variant.

In addition to the impact on pathogen and commensal binding to epithelia, the DE and network analyses suggest that the *FUT2* c.461G>A variant also has a downstream effect on basic cellular pathways (**Figures 2**, **3** and **Tables 4**, **7**). For example, *FN1* is a modulator of ME anti-inflammatory response (Song et al., 2013) as well as a binding site for otopathogen *Staphylococcus aureus* (Fowler et al., 2000) and group A *Streptococcus* (McNitt et al., 2018). FN1 protein expression was also previously demonstrated to be dysregulated by viral infection (Simon et al., 2015; Qiao et al., 2021); however, viruses are not included in this study due to sample collection methods. Notably we only observed a nominal increase in *Staphylococcus* abundance in the NP (but not ME) of carriers of the *RASIP1* variant (**Figure 7**), but not in carriers of the *FUT2* variant (**Figure 6**). In addition, NTHi inoculation of mouse ME resulted in upregulation of *Fn1* (**Figure 4** and **Table 3**). In contrast, in our OM patients with the *FUT2* stop variant, *FN1* was downregulated (**Figure 2** and **Table 4**), indicating that non-functional *FUT2* might also affect the direction of regulation of the immune network that includes *FN1* and also *RASIP1, MTAP* and *MLL2/KMT2D* (**Figure 3**). It should be noted that *KMT2D* variants are responsible for Kabuki Syndrome which is characterized by increased rates of OM as well as other immunological abnormalities (Hoffman et al., 2005; Ng et al., 2010; Yap et al., 2020; Boniel et al., 2021).

**Table 7:** Summary of relevant knowledge of *FUT2*, *RASIP1* and DE genes.

Gene	Prior findings in literature
*FUT2* (alpha-[1,2]-fucosyltransferase), MIM 182100	c.461G>A variant confers non-secretor status of ABO(H) antigens on mucosal epithelia (Magalhaes et al., 2016) Non-secretors demonstrate decreased commensal load allowing an increase in bacterial pathogen colonization (Giese et al., 2020) Non-secretor status affects mucus barrier (Magalhaes et al., 2016)
*RASIP1* (Ras interacting protein 1), MIM 609623	Crucial to formation of vascular structures *via* angiogenesis and vasculogenesis (Xu et al., 2009) Involved in endothelial barrier function (Xu et al., 2011) Expressed in middle ear endothelial cells (Ryan et al., 2020)
*FN1* (fibronectin-1), MIM 135600	Glycoprotein found in extracellular matrix and on cell surface (McDonald et al., 1982; Woods et al., 1986) Involved in cell adhesion, migration, host defense and wound healing (McAuslan et al., 1980; Clark et al., 1982; Hill et al., 1984; Woods et al., 1986) Expressed in human middle ear epithelial cells & identified as a key modulator of anti-inflammatory response to extracellular stress (Song et al., 2013) Utilized by *S. aureus* to gain entry to host cells (Fowler et al., 2000)
*KMT2D/MLL2* (histone-lysine N-methyltransferase 2B; myeloid/lymphoid or mixed-lineage leukemia protein 2), MIM 602113	*KMT2D* mutations are the cause of the majority of cases of Kabuki syndrome (KS; MIM 147920) (Ng et al., 2010; Yap et al., 2020) KS patients have high rate of infections and array of immunological abnormalities (Hoffman et al., 2005) OM occurs in 55-90% of KS patients (Boniel et al., 2021)
*MTAP* (S-methyl-5’-thioadenosine phosphorylase)	* *Mtap^+/-^* mice had no hearing loss, while *Mtap^-/-^* was embryonic lethal (Williamson et al., 2007)
*MUC16* (cell-surface associated mucin 16)	Transmembrane mucin expressed in human and mouse middle ear and airway epithelia (Kerschner, 2007; Kerschner et al., 2010) Contributes to composition of mucous barrier as part of host defense against infection (Kesimer et al., 2009) Upregulated in middle ear epithelia of OM patients as compared to normal controls (Stabenau et al., 2021)

In conclusion, we propose that the mechanistic effects of the *FUT2* c.461G>A variant on OM susceptibility are two-fold: (1) Non-secretor status conferred by this *FUT2* stop variant alters the profiles of bacterial taxa that bind to ME and NP mucosal epithelia and thereby increases susceptibility to bacterial infection in mucosal epithelia; and (2) *FUT2* variants affect expression of genes including downregulation of *MUC16* and those connected to an immune network, which leads to further susceptibility to infection as well as impaired immune responses (**Figure 3**) and basic cellular processes (**Table 2**) within the ME mucosal epithelium. Through increased understanding of the effects of pathogenic variants on dysbiosis and gene regulation in OM, the ability to determine risk for patients due to specific genetic variants may be improved, and thereafter enhance prevention and treatment protocols for OM using more targeted antibiotics for otopathogens associated with these variants.

## Data Availability Statement

The datasets presented in this study can be found in online repositories. The names of the repository/repositories and accession number(s) can be found below: https://www.ncbi.nlm.nih.gov/gap/, phs001941.v1.p1 https://www.ncbi.nlm.nih.gov/sra/, BioProject ID PRJNA748418.

## Ethics Statement

The studies involving human participants were reviewed and approved by Colorado Multiple Institutional Review Board. Written informed consent to participate in this study was provided by the participants’ legal guardian/next of kin. The animal study was reviewed and approved by Institutional Animal Care and Use Committee Veterans Affairs Medical Center, San Diego, California.

## Author Contributions

RS-C conceptualized the study. MS, S-OS, TW, PY, SG, SC, HJ, JP, KC, and NF recruited patients and collected samples. TB performed isolation of human DNA and RNA and microbial DNA samples and submitted them for sequencing. CE and EL performed RNA sequence analyses. CE performed network analyses. JK, CR, and DF performed 16S rRNA sequencing. CE and DF performed analyses of microbiota data. AR performed mouse expression studies. CE, AR, and RS-C wrote the manuscript. All authors read and approved the manuscript.

## Funding

This work was supported by the National Institutes of Health (NIH) - National Institute on Deafness and Other Communication Disorders (NIDCD) *via* grant R01 DC015004 (to RS-C). Mouse studies were supported by grant R01 DC000129 from NIH-NIDCD (to AR). CE was supported by the T32 DC012280 grant from NIH-NIDCD (to Sue C. Kinnamon and HJ). The contents of this manuscript are solely the responsibility of the authors and do not necessarily represent the official views of the NIH.

## Conflict of Interest

AR is a cofounder of Otonomy, Inc., serves as a consultant and member of the Scientific Advisory Board, and holds an equity position in the company. The UCSD and San Diego VA Committees on Conflict of Interest have approved this relationship. Otonomy, Inc. had no role in the conduct of this study and the writing of the manuscript.

The remaining authors declare that the research was conducted in the absence of any commercial or financial relationships that could be construed as a potential conflict of interest.

## Publisher’s Note

All claims expressed in this article are solely those of the authors and do not necessarily represent those of their affiliated organizations, or those of the publisher, the editors and the reviewers. Any product that may be evaluated in this article, or claim that may be made by its manufacturer, is not guaranteed or endorsed by the publisher.
