# Supplementary material for: The FUT2 Variant c.461G>A (p.Trp154*) Is Associated With Differentially Expressed Genes and Nasopharyngeal Microbiota Shifts in Patients With Otitis Media
Source: Front Cell Infect Microbiol. 2022 Jan 14;11:798246. doi: 10.3389/fcimb.2021.798246 (PMC8798324; doi:10.3389/fcimb.2021.798246)
Supplement: Supplementary file 1 [file DataSheet_1.pdf]

Supplementary Material

**Supplementary Table 1. α-diversity indices in the middle ear and nasopharynx by *FUT2* c.461G>A carriage**		
α-diversity indices	*p-value, all ethnicities*	*p-value, White only*
Middle Ear	n=34	n=23
Chao1	0.03	0.45
Shannon Diversity (*H*)	0.15	0.59
Shannon Evenness (H/H_{max})	0.19	0.59
Nasopharynx	n=65	n=40
Chao1	0.36	0.33
Shannon Diversity (*H*)	0.50	0.25
Shannon Evenness (H/H_{max})	0.61	0.24

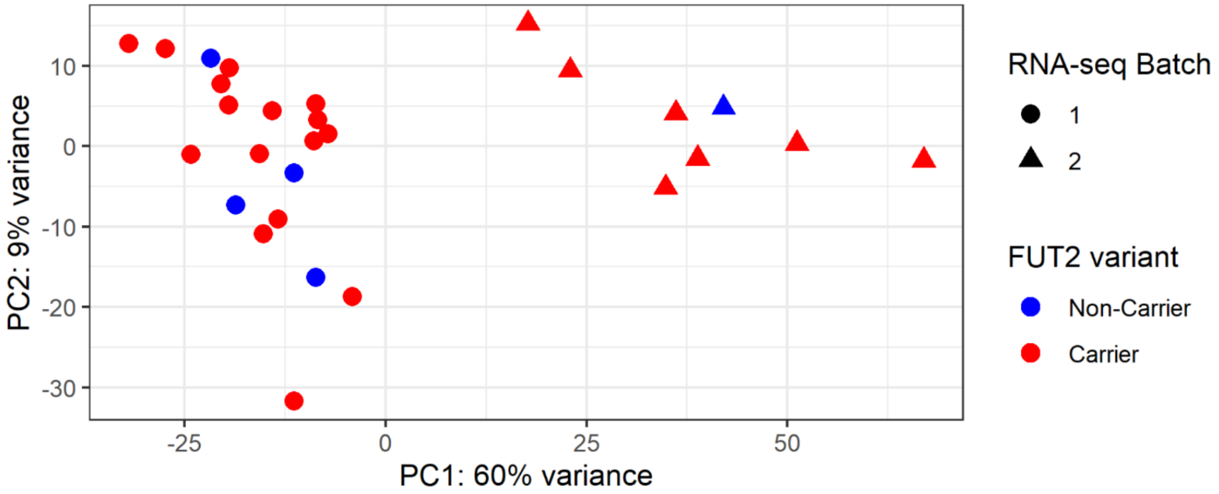

Supplementary Figure 1. Principal components analysis of RNA-seq samples. PCA revealed that PC1 accounts for 60% of variance in the sample set and results in clustering by batch (batch 1 n=20, batch 2 n=8)

Supplementary Table 2. List of taxa shown in *FUT2* microbiome analyses in Figure 6c-6d

	Prevalence				Mean abundance (s.d.)				
**Taxon**	**ME carrier**	**ME non-carrier**	**NP carrier**	**NP non-carrier**	**ME carrier**	**ME non-carrier**	**NP carrier**	**NP non-carrier**	**Phylum**
Acti:Actinomyces	73.1	37.5	94.1	100	0.2 (0.6)	0.0 (0.0)	0.3 (0.5)	0.8 (1.8)	Actinobacteria
Acti:Corynebacteriaceae	73.1	25	43.1	28.6	0.1 (0.3)	0.0 (0.0)	0.0 (0.0)	0.0 (0.0)	Actinobacteria
Acti:Corynebacterium	96.2	75	94.1	100	1.7 (2.4)	0.7 (1.5)	3.1 (11.2)	0.9 (2.2)	Actinobacteria
Acti:Kineococcus	42.3	25	33.3	14.3	0.4 (1.3)	0.0 (0.0)	0.0 (0.1)	0.0 (0.1)	Actinobacteria
Acti:Propionibacteriaceae	15.4	12.5	19.6	21.4	0.1 (0.3)	0.0 (0.0)	0.0 (0.0)	0.0 (0.0)	Actinobacteria
Acti:Propionibacterium	100	75	94.1	78.6	2.2 (4.7)	0.6 (1.5)	0.3 (0.9)	0.1 (0.1)	Actinobacteria
Acti:Rothia	69.2	37.5	90.2	100	0.2 (0.4)	0.0 (0.0)	0.5 (1.5)	0.3 (0.5)	Actinobacteria
Acti:Turicella	38.5	12.5	17.6	21.4	0.1 (0.4)	0.0 (0.0)	0.0 (0.0)	0.0 (0.0)	Actinobacteria
Bact:Bergeyella	30.8	25	84.3	78.6	0.1 (0.2)	0.0 (0.0)	0.1 (0.2)	0.1 (0.1)	Bacteroidetes
Bact:Capnocytophaga	38.5	25	76.5	78.6	0.0 (0.1)	0.0 (0.0)	0.2 (0.5)	0.1 (0.2)	Bacteroidetes
Bact:Porphyromonas	80.8	62.5	98	100	0.5 (1.0)	0.0 (0.0)	1.0 (1.9)	0.5 (0.6)	Bacteroidetes
Bact:Prevotella	76.9	87.5	98	100	0.8 (2.1)	0.0 (0.0)	2.8 (3.9)	2.0 (2.6)	Bacteroidetes
Bact:S24-7	26.9	25	15.7	14.3	0.2 (0.9)	0.0 (0.0)	0.0 (0.0)	0.0 (0.0)	Bacteroidetes
Bact:Tannerella	3.8	12.5	29.4	42.9	0.0 (0.1)	0.0 (0.0)	0.2 (0.9)	0.0 (0.1)	Bacteroidetes
Candidate-division-SR1	3.8	0	29.4	57.1	0.0 (0.0)	0.0 (0.0)	0.0 (0.1)	0.1 (0.3)	Candidate-division-SR1
Candidate-division-TM7	34.6	25	41.2	92.9	0.0 (0.1)	0.0 (0.0)	0.0 (0.1)	0.2 (0.4)	Candidate-division-TM7
Cyan:Chloroplast	73.1	50	70.6	50	0.3 (0.4)	0.1 (0.2)	0.1 (0.6)	0.1 (0.2)	Cyanobacteria
Firm:Abiotrophia	19.2	0	60.8	64.3	0.0 (0.0)	0.0 (0.0)	0.1 (0.2)	0.2 (0.3)	Firmicutes
Firm:Alloiococcus	73.1	37.5	41.2	14.3	8.5 (20.6)	0.7 (2.0)	0.0 (0.0)	0.0 (0.0)	Firmicutes
Firm:Anaerococcus	50	25	47.1	35.7	0.1 (0.3)	0.0 (0.0)	0.0 (0.0)	0.0 (0.0)	Firmicutes
Firm:Anoxybacillus	26.9	37.5	9.8	14.3	0.0 (0.1)	0.2 (0.5)	0.0 (0.0)	0.0 (0.0)	Firmicutes
Firm:Bacilli	46.2	25	100	100	0.4 (2.0)	0.0 (0.0)	0.4 (0.7)	0.4 (0.6)	Firmicutes

Firm:Bacillus	88.5	62.5	70.6	64.3	0.8 (1.6)	0.2 (0.4)	0.1 (0.3)	0.0 (0.0)	Firmicutes
Firm:Dolosigranulum	61.5	37.5	76.5	50	0.5 (1.8)	0.0 (0.1)	3.1 (8.9)	3.6 (10.5)	Firmicutes
Firm:Finegoldia	50	25	47.1	35.7	0.1 (0.2)	0.0 (0.0)	0.0 (0.1)	0.0 (0.0)	Firmicutes
Firm:Gemella	65.4	37.5	96.1	100	0.2 (0.8)	0.0 (0.0)	1.4 (3.0)	2.1 (2.7)	Firmicutes
Firm:Geobacillus	19.2	0	3.9	7.1	0.1 (0.3)	0.0 (0.0)	0.0 (0.0)	0.0 (0.0)	Firmicutes
Firm:Johnsonella	3.8	12.5	64.7	64.3	0.0 (0.0)	0.0 (0.0)	0.1 (0.3)	0.9 (2.8)	Firmicutes
Firm:Lachnospiraceae	50	37.5	62.7	57.1	0.1 (0.4)	0.0 (0.0)	0.0 (0.1)	0.0 (0.1)	Firmicutes
Firm:Lactobacillales	65.4	25	94.1	100	0.2 (0.5)	0.0 (0.0)	0.7 (1.2)	0.6 (0.8)	Firmicutes
Firm:Lactobacillus	80.8	37.5	54.9	57.1	1.9 (4.2)	0.6 (1.3)	0.0 (0.1)	0.1 (0.3)	Firmicutes
Firm:Lactococcus	26.9	0	54.9	50	0.0 (0.1)	0.0 (0.0)	0.1 (0.2)	0.0 (0.0)	Firmicutes
Firm:Parvimonas	23.1	25	68.6	57.1	0.2 (0.9)	0.0 (0.0)	0.4 (1.8)	0.2 (0.7)	Firmicutes
Firm:Peptostreptococcaceae	11.5	25	66.7	85.7	0.0 (0.0)	0.0 (0.0)	0.2 (0.6)	0.0 (0.1)	Firmicutes
Firm:Selenomonas	0	25	43.1	78.6	0.0 (0.0)	0.0 (0.0)	0.1 (0.2)	0.0 (0.1)	Firmicutes
Firm:Staphylococcus	96.2	75	94.1	78.6	8.4 (19.7)	9.3 (17.6)	2.3 (13.9)	12.9 (28.4)	Firmicutes
Firm:Streptococcus	100	100	100	100	10.0 (21.0)	2.2 (5.9)	15.7 (18.8)	11.6 (13.6)	Firmicutes
Firm:Turicibacter	11.5	12.5	2	0	0.1 (0.2)	0.0 (0.0)	0.0 (0.0)	0.0 (0.0)	Firmicutes
Firm:Veillonella	76.9	50	96.1	100	0.4 (1.0)	0.0 (0.0)	1.8 (4.3)	1.2 (1.4)	Firmicutes
Firmicutes	30.8	12.5	58.8	64.3	0.0 (0.2)	0.0 (0.0)	0.0 (0.1)	0.0 (0.0)	Firmicutes
Fuso:Fusobacteriales	23.1	0	58.8	78.6	0.0 (0.0)	0.0 (0.0)	0.2 (0.6)	0.2 (0.3)	Fusobacteria
Fuso:Fusobacterium	80.8	62.5	98	100	0.2 (0.4)	0.0 (0.0)	2.7 (7.2)	1.5 (2.3)	Fusobacteria
Fuso:Leptotrichia	38.5	50	76.5	85.7	0.1 (0.2)	0.0 (0.0)	0.2 (0.5)	0.3 (0.5)	Fusobacteria
Fuso:Leptotrichiaceae	11.5	0	72.5	85.7	0.0 (0.1)	0.0 (0.0)	0.2 (0.4)	0.3 (0.8)	Fusobacteria
Prot:Acinetobacter	69.2	37.5	58.8	64.3	0.3 (0.8)	0.0 (0.1)	0.0 (0.1)	0.0 (0.0)	Proteobacteria
Prot:Actinobacillus	34.6	37.5	51	85.7	0.2 (0.5)	0.0 (0.0)	0.6 (2.3)	1.6 (3.1)	Proteobacteria
Prot:Alcaligenaceae	38.5	50	54.9	35.7	0.2 (1.1)	0.0 (0.0)	0.0 (0.0)	0.0 (0.0)	Proteobacteria
Prot:Brevundimonas	30.8	25	33.3	7.1	0.2 (0.8)	0.0 (0.0)	0.0 (0.0)	0.0 (0.0)	Proteobacteria

Prot:Campylobacter	34.6	37.5	64.7	78.6	0.0 (0.0)	0.0 (0.0)	0.1 (0.3)	0.1 (0.1)	Proteobacteria
Prot:Comamonadaceae	76.9	50	45.1	28.6	0.3 (0.8)	0.0 (0.0)	0.0 (0.1)	0.0 (0.0)	Proteobacteria
Prot:Eikenella	15.4	0	52.9	50	0.0 (0.0)	0.0 (0.0)	0.2 (0.8)	0.0 (0.0)	Proteobacteria
Prot:Enterobacter	84.6	62.5	54.9	42.9	0.2 (0.4)	0.3 (0.7)	0.1 (0.3)	0.0 (0.0)	Proteobacteria
Prot:Enterobacteriaceae	76.9	37.5	72.5	71.4	0.8 (3.9)	0.1 (0.1)	0.0 (0.2)	0.0 (0.0)	Proteobacteria
Prot:Escherichia-Shigella	96.2	62.5	90.2	78.6	1.1 (1.8)	0.8 (2.3)	0.4 (2.0)	0.0 (0.1)	Proteobacteria
Prot:Gammaproteobacteria	92.3	75	80.4	78.6	3.0 (6.1)	1.1 (2.8)	0.3 (1.3)	0.0 (0.0)	Proteobacteria
Prot:Haemophilus	100	100	100	100	40.1 (45.1)	79.0 (34.5)	33.5 (35.2)	36.3 (35.9)	Proteobacteria
Prot:Kingella	15.4	0	47.1	50	0.0 (0.0)	0.0 (0.0)	0.0 (0.2)	0.0 (0.1)	Proteobacteria
Prot:mitochondria	65.4	37.5	39.2	42.9	0.1 (0.1)	0.0 (0.1)	0.1 (0.3)	0.0 (0.0)	Proteobacteria
Prot:Moraxella	96.2	100	96.1	100	8.4 (22.3)	1.2 (3.1)	19.4 (27.6)	12.5 (18.8)	Proteobacteria
Prot:Neisseria	84.6	87.5	98	100	0.2 (0.5)	0.0 (0.0)	3.9 (10.0)	5.2 (7.1)	Proteobacteria
Prot:Neisseriaceae	42.3	37.5	68.6	71.4	0.1 (0.4)	0.0 (0.0)	0.3 (1.2)	0.0 (0.1)	Proteobacteria
Prot:Pseudomonadales	50	37.5	39.2	14.3	0.1 (0.3)	0.0 (0.0)	0.0 (0.0)	0.0 (0.0)	Proteobacteria
Prot:Pseudomonas	76.9	37.5	74.5	64.3	0.2 (0.6)	0.0 (0.1)	0.0 (0.1)	0.0 (0.0)	Proteobacteria
Prot:Rhodobacteraceae	26.9	12.5	11.8	28.6	0.8 (4.0)	0.0 (0.0)	0.0 (0.0)	0.0 (0.0)	Proteobacteria
Prot:Sphingobium	15.4	0	7.8	7.1	0.1 (0.4)	0.0 (0.0)	0.0 (0.0)	0.0 (0.0)	Proteobacteria
Spir:Treponema	15.4	12.5	45.1	28.6	0.0 (0.1)	0.0 (0.0)	0.6 (3.1)	0.0 (0.1)	Spirochaetae

Supplementary Table 3. List of taxa shown in *RASIP1* microbiome analyses in Figure 7c-7d

	**Prevalence**				**Mean abundance (s.d.)**				
**Taxon**	**ME carrier**	**ME non-carrier**	**NP carrier**	**NP non-carrier**	**ME carrier**	**ME non-carrier**	**NP carrier**	**NP non-carrier**	**Phylum**
Acti:Actinomyces	73.1	37.5	96	93.3	0.2 (0.6)	0.0 (0.0)	0.3 (0.5)	0.7 (1.8)	Actinobacteria
Acti:Corynebacteriaceae	69.2	37.5	46	20	0.1 (0.3)	0.0 (0.0)	0.0 (0.0)	0.0 (0.0)	Actinobacteria
Acti:Corynebacterium	96.2	75	94	100	1.7 (2.4)	0.7 (1.5)	3.1 (11.3)	0.8 (2.2)	Actinobacteria
Acti:Kineococcus	42.3	25	36	6.7	0.4 (1.3)	0.0 (0.0)	0.0 (0.1)	0.0 (0.0)	Actinobacteria
Acti:Propionibacteriaceae	15.4	12.5	20	20	0.1 (0.3)	0.0 (0.0)	0.0 (0.0)	0.0 (0.0)	Actinobacteria
Acti:Propionibacterium	100	75	94	80	2.2 (4.7)	0.6 (1.5)	0.3 (0.9)	0.0 (0.0)	Actinobacteria
Acti:Rothia	65.4	50	90	100	0.2 (0.4)	0.0 (0.0)	0.5 (1.5)	0.3 (0.5)	Actinobacteria
Acti:Turicella	38.5	12.5	16	26.7	0.1 (0.4)	0.0 (0.0)	0.0 (0.0)	0.0 (0.0)	Actinobacteria
Bact:Bergeyella	34.6	12.5	82	86.7	0.1 (0.2)	0.0 (0.0)	0.1 (0.2)	0.1 (0.2)	Bacteroidetes
Bact:Capnocytophaga	38.5	25	74	86.7	0.0 (0.1)	0.0 (0.0)	0.2 (0.5)	0.2 (0.3)	Bacteroidetes
Bact:Porphyromonas	80.8	62.5	98	100	0.5 (1.0)	0.0 (0.0)	1.0 (1.9)	0.6 (0.7)	Bacteroidetes
Bact:Prevotella	76.9	87.5	98	100	0.8 (2.1)	0.0 (0.0)	2.9 (3.9)	1.8 (2.3)	Bacteroidetes
Bact:S24-7	26.9	25	14	20	0.2 (0.9)	0.0 (0.0)	0.0 (0.0)	0.0 (0.0)	Bacteroidetes
Bact:Tannerella	3.8	12.5	28	46.7	0.0 (0.1)	0.0 (0.0)	0.2 (0.9)	0.0 (0.1)	Bacteroidetes
Candidate-division-SR1	3.8	0	26	66.7	0.0 (0.0)	0.0 (0.0)	0.0 (0.1)	0.1 (0.3)	Candidate-division-SR1
Candidate-division-TM7	34.6	25	42	86.7	0.0 (0.1)	0.0 (0.0)	0.0 (0.1)	0.2 (0.4)	Candidate-division-TM7
Cyan:Chloroplast	73.1	50	74	40	0.3 (0.4)	0.1 (0.2)	0.1 (0.6)	0.0 (0.0)	Cyanobacteria
Firm:Abiotrophia	19.2	0	64	53.3	0.0 (0.0)	0.0 (0.0)	0.1 (0.2)	0.1 (0.3)	Firmicutes
Firm:Alloiococcus	73.1	37.5	42	13.3	8.4 (20.6)	0.8 (2.0)	0.0 (0.0)	0.0 (0.0)	Firmicutes
Firm:Anaerococcus	46.2	37.5	54	13.3	0.1 (0.3)	0.0 (0.0)	0.0 (0.0)	0.0 (0.0)	Firmicutes
Firm:Anoxybacillus	30.8	25	12	6.7	0.0 (0.1)	0.2 (0.5)	0.0 (0.0)	0.0 (0.0)	Firmicutes

Firm:Bacilli	42.3	37.5	100	100	0.4 (2.0)	0.0 (0.0)	0.4 (0.7)	0.4 (0.6)	Firmicutes
Firm:Bacillus	88.5	62.5	72	60	0.8 (1.6)	0.2 (0.4)	0.1 (0.3)	0.0 (0.0)	Firmicutes
Firm:Dolosigranulum	61.5	37.5	78	46.7	0.5 (1.8)	0.0 (0.1)	3.2 (8.9)	3.4 (10.1)	Firmicutes
Firm:Finegoldia	50	25	44	46.7	0.1 (0.2)	0.0 (0.0)	0.0 (0.1)	0.0 (0.0)	Firmicutes
Firm:Gemella	61.5	50	96	100	0.2 (0.8)	0.0 (0.0)	1.4 (3.0)	2.4 (2.6)	Firmicutes
Firm:Geobacillus	19.2	0	4	6.7	0.1 (0.3)	0.0 (0.0)	0.0 (0.0)	0.0 (0.0)	Firmicutes
Firm:Johnsonella	3.8	12.5	62	73.3	0.0 (0.0)	0.0 (0.0)	0.3 (1.5)	0.2 (0.3)	Firmicutes
Firm:Lachnospiraceae	50	37.5	64	53.3	0.1 (0.4)	0.0 (0.0)	0.0 (0.1)	0.0 (0.1)	Firmicutes
Firm:Lactobacillales	61.5	37.5	94	100	0.2 (0.5)	0.0 (0.0)	0.7 (1.2)	0.6 (0.8)	Firmicutes
Firm:Lactobacillus	80.8	37.5	54	60	1.9 (4.2)	0.6 (1.3)	0.0 (0.1)	0.1 (0.3)	Firmicutes
Firm:Lactococcus	26.9	0	58	40	0.0 (0.1)	0.0 (0.0)	0.1 (0.2)	0.0 (0.0)	Firmicutes
Firm:Parvimonas	23.1	25	66	66.7	0.2 (0.9)	0.0 (0.0)	0.5 (1.8)	0.2 (0.7)	Firmicutes
Firm:Peptostreptococcaceae	11.5	25	70	73.3	0.0 (0.0)	0.0 (0.0)	0.2 (0.6)	0.0 (0.1)	Firmicutes
Firm:Selenomonas	0	25	44	73.3	0.0 (0.0)	0.0 (0.0)	0.1 (0.2)	0.0 (0.1)	Firmicutes
Firm:Staphylococcus	96.2	75	94	80	8.4 (19.7)	9.3 (17.6)	3.7 (17.1)	7.2 (22.2)	Firmicutes
Firm:Streptococcaceae	30.8	12.5	80	86.7	0.0 (0.1)	0.0 (0.0)	0.2 (0.5)	0.0 (0.1)	Firmicutes
Firm:Streptococcus	100	100	100	100	10.0 (21.0)	2.2 (5.9)	16.5 (19.5)	9.3 (8.1)	Firmicutes
Firm:Turicibacter	11.5	12.5	0	6.7	0.1 (0.2)	0.0 (0.0)	0.0 (0.0)	0.0 (0.0)	Firmicutes
Firm:Veillonella	73.1	62.5	96	100	0.4 (1.0)	0.0 (0.0)	1.8 (4.3)	1.0 (1.3)	Firmicutes
Firmicutes	30.8	12.5	62	53.3	0.0 (0.2)	0.0 (0.0)	0.0 (0.1)	0.0 (0.0)	Firmicutes
Fuso:Fusobacteriales	23.1	0	60	73.3	0.0 (0.0)	0.0 (0.0)	0.2 (0.6)	0.1 (0.3)	Fusobacteria
Fuso:Fusobacterium	80.8	62.5	98	100	0.2 (0.4)	0.0 (0.0)	2.8 (7.3)	1.4 (2.2)	Fusobacteria
Fuso:Leptotrichia	38.5	50	74	93.3	0.1 (0.2)	0.0 (0.0)	0.2 (0.5)	0.4 (0.5)	Fusobacteria
Fuso:Leptotrichiaceae	11.5	0	70	93.3	0.0 (0.1)	0.0 (0.0)	0.1 (0.4)	0.4 (0.8)	Fusobacteria
Prot:Acinetobacter	69.2	37.5	60	60	0.3 (0.8)	0.0 (0.1)	0.0 (0.1)	0.0 (0.0)	Proteobacteria
Prot:Actinobacillus	34.6	37.5	50	86.7	0.2 (0.5)	0.0 (0.0)	0.6 (2.3)	1.6 (3.0)	Proteobacteria

Prot:Alcaligenaceae	38.5	50	54	40	0.2 (1.1)	0.0 (0.0)	0.0 (0.0)	0.0 (0.0)	Proteobacteria
Prot:Brevundimonas	30.8	25	34	6.7	0.2 (0.8)	0.0 (0.0)	0.0 (0.0)	0.0 (0.0)	Proteobacteria
Prot:Campylobacter	30.8	50	66	73.3	0.0 (0.0)	0.0 (0.0)	0.1 (0.3)	0.1 (0.2)	Proteobacteria
Prot:Comamonadaceae	76.9	50	48	20	0.3 (0.8)	0.0 (0.0)	0.0 (0.1)	0.0 (0.0)	Proteobacteria
Prot:Eikenella	15.4	0	52	53.3	0.0 (0.0)	0.0 (0.0)	0.2 (0.8)	0.0 (0.0)	Proteobacteria
Prot:Enterobacter	80.8	75	52	53.3	0.2 (0.4)	0.3 (0.7)	0.1 (0.3)	0.0 (0.0)	Proteobacteria
Prot:Enterobacteriaceae	76.9	37.5	72	73.3	0.8 (3.9)	0.1 (0.1)	0.1 (0.2)	0.0 (0.0)	Proteobacteria
Prot:Escherichia-Shigella	96.2	62.5	90	80	1.1 (1.8)	0.8 (2.3)	0.4 (2.0)	0.0 (0.0)	Proteobacteria
Prot:Gammaproteobacteria	92.3	75	82	73.3	3.0 (6.1)	1.1 (2.8)	0.3 (1.3)	0.0 (0.0)	Proteobacteria
Prot:Haemophilus	100	100	100	100	40.1 (45.1)	78.9 (34.4)	33.6 (36.3)	36.0 (32.0)	Proteobacteria
Prot:Kingella	15.4	0	42	66.7	0.0 (0.0)	0.0 (0.0)	0.0 (0.2)	0.0 (0.1)	Proteobacteria
Prot:mitochondria	65.4	37.5	42	33.3	0.1 (0.1)	0.0 (0.1)	0.1 (0.3)	0.0 (0.0)	Proteobacteria
Prot:Moraxella	96.2	100	96	100	8.4 (22.3)	1.2 (3.1)	16.8 (26.8)	21.6 (23.8)	Proteobacteria
Prot:Neisseria	84.6	87.5	98	100	0.2 (0.5)	0.0 (0.0)	3.7 (10.1)	5.8 (6.7)	Proteobacteria
Prot:Neisseriaceae	42.3	37.5	68	73.3	0.1 (0.4)	0.0 (0.0)	0.3 (1.2)	0.0 (0.1)	Proteobacteria
Prot:Pseudomonadales	50	37.5	36	26.7	0.1 (0.3)	0.0 (0.0)	0.0 (0.0)	0.0 (0.0)	Proteobacteria
Prot:Pseudomonas	76.9	37.5	74	66.7	0.2 (0.6)	0.0 (0.1)	0.0 (0.1)	0.0 (0.0)	Proteobacteria
Prot:Rhodobacteraceae	26.9	12.5	18	6.7	0.8 (4.0)	0.0 (0.0)	0.0 (0.0)	0.0 (0.0)	Proteobacteria
Prot:Rhodobium	3.8	0	4	6.7	0.0 (0.0)	0.0 (0.0)	0.0 (0.0)	0.0 (0.0)	Proteobacteria

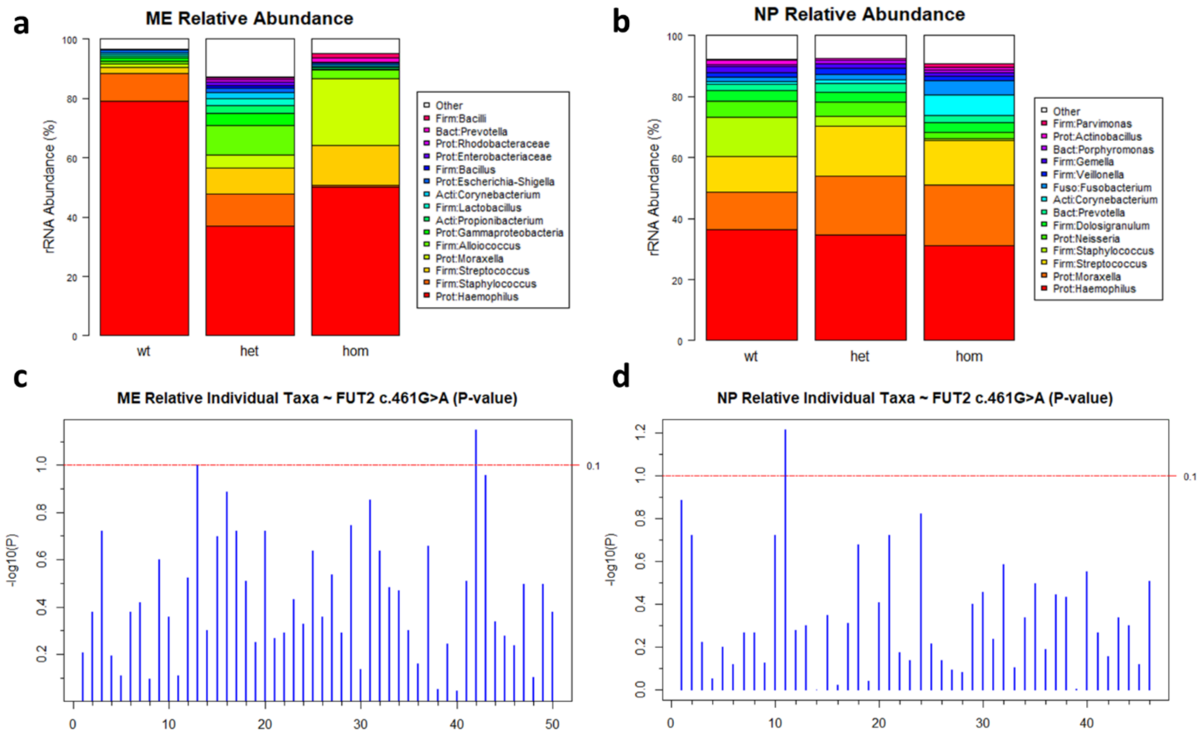

Supplementary Figure 2. Relative abundance of individual taxa in the middle ears (ME) and nasopharynges (NP) by *FUT2* c.461G>A variant genotype. (a) Cumulative relative abundance profiles in the ME of individuals that are wildtype (n=6), heterozygous (n=16) and homozygous (n=5) for *FUT2* c.461G>A variant. (b) Cumulative relative abundance profiles in the NP of individuals that are wildtype (n=14), heterozygous (n=34) and homozygous (n=17) for *FUT2* c.461G>A variant. Plots showing *p*-values for relative abundance of individual bacterial taxa in the (c) ME and (d) NP of non-carriers versus carriers after adjusting for batch. *Dashed lines* indicate significance thresholds where the *red line* is unadjusted-*p*=0.1. There were no significant taxa for the ME or NP when individuals were grouped by genotype

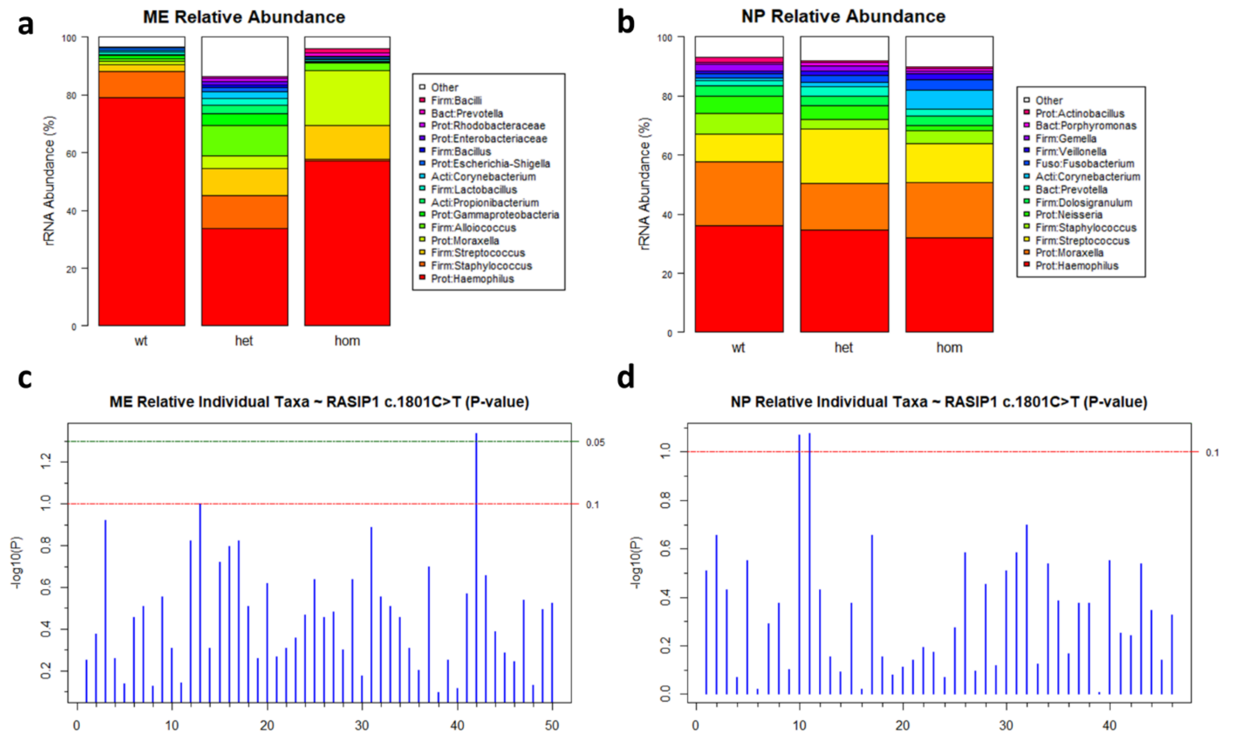

Supplementary Figure 3. Relative abundance of individual taxa in the ME and NP of carriers and non-carriers of the *RASIP1* c.1801C>T variant. (a) Cumulative relative abundance profiles in the ME of individuals that are wildtype (n=6), heterozygous (n=15) and homozygous (n=6) for *RASIP1* c.1801C>T variant. (b) Cumulative relative abundance profiles in the NP of individuals that are wildtype (n=6), heterozygous (n=18) and homozygous (n=9) for *RASIP1* c.1801C>T variant. Plots showing *p*-values for relative abundance of individual bacterial taxa in the (c) ME and (d) NP of non-carriers versus carriers after adjusting for batch. *Dashed lines* indicate significance thresholds where the *red line* is unadjusted-*p*=0.1 (non-significant) and the *green line* indicates unadjusted-*p*=0.05. In the ME, *Haemophilus* was nominally associated with wildtype genotype. There were no significant taxa for the NP.
